# Extracellular Vesicles Derived From *Streptococcus anginosus* Aggravate Lupus Nephritis by Triggering TLR2‐MyD88‐NF‐κB Signalling in NK Cells

**DOI:** 10.1002/jev2.70134

**Published:** 2025-07-17

**Authors:** Ying Gong, Lingyue Jin, Lina Duan, Jie Xiao, Yao Li, HongXia Wang, Haifang Wang, Wanying Lin, Yi Zhang, Xiufeng Gan, Shuyin Pang, Yurong Qiu, Weinan Lai, Lei Zheng, Haixia Li

**Affiliations:** ^1^ Department of Laboratory Medicine, Guangdong Provincial Key Laboratory of Precision Medical Diagnostics, Guangdong Engineering and Technology Research Center for Rapid Diagnostic Biosensors, Guangdong Provincial Key Laboratory of Single‐cell and Extracellular Vesicles, Nanfang Hospital Southern Medical University Guangzhou P.R. China; ^2^ Guangdong Provincial Clinical Research Center for Laboratory Medicine, Nanfang Hospital Southern Medical University Guangzhou P.R. China; ^3^ Department of Internal Medicine, Division of Hematology University of Maastricht Maastricht the Netherlands; ^4^ Department of Laboratory Medicine, Foshan Hospital of Traditional Chinese Medicine Guangzhou University of Chinese Medicine Foshan P.R. China; ^5^ Huayin Medical Laboratory Center Co., Ltd Guangzhou P.R. China; ^6^ Department of Rheumatology and Immunology, Nanfang Hospital Southern Medical University Guangzhou P.R. China; ^7^ State Key Laboratory of Multi‐organ Injury Prevention and Treatment, Nanfang Hospital Southern Medical University Guangzhou P.R. China

**Keywords:** bacterial extracellular vesicle, lupus nephritis (LN), natural killer cells (NK), systemic lupus erythematosus (SLE), TLR2‐ MyD88‐NF‐κB signalling

## Abstract

Systemic lupus erythematosus (SLE) has been linked to gut microbiome dysbiosis, notably an overabundance of *Streptococcus anginosus*; however, the impact of this microbial imbalance on disease pathogenesis remains unclear. Here, we investigated the contribution of *S. anginosus*‐derived extracellular vesicles (*SA*‐EVs) to SLE progression, with an emphasis on lupus nephritis (LN). Fifty‐four SLE patients and 43 healthy controls (HC) were recruited. The faecal, blood and serum samples from participants were collected. SLE disease activity (SLEDA) was evaluated by the SLEDA Index (SLEDAI). Stool *S. anginosus* abundance was quantified by quantitative PCR, NK cell activation by flow cytometry and serum proinflammatory cytokines profile by ELISA. Lupus‐prone MRL/lpr mice were orally administered *SA*‐EVs to evaluate in vivo inflammatory responses, renal NK cell activation and renal histopathological changes. *S. anginosus* levels were significantly elevated in SLE patients relative to HC, positively correlated with SLEDAI scores and NK cell cytotoxicity. In vitro, *SA*‐EVs stimulation of patient NK cells significantly heightened proinflammatory mediator production (granzyme B, TNF‐α), increased cytotoxicity and downregulated inhibitory receptors (TIM‐3, NKG2A, TIGIT) compared to control EVs from *S. Salivarius* (*SS*‐EVs). Mechanistically, lipoteichoic acid (LTA) within *SA*‐EVs engaged Toll‐like receptor 2 (TLR2) on NK cells, activating MyD88/NF‐κB signalling pathway. In MRL/lpr mice, *SA*‐EVs treatment increased renal immune complex deposition, upregulated renal NK cell activation markers (NKp44, NKp46), and exacerbated LN pathology with greater immune cell infiltration and inflammatory cytokine levels. Furthermore, NK cell depletion with anti‐NK1.1 antibodies significantly prolonged survival in *SA*‐EVs administered mice. Thus, *SA*‐EVs exacerbate SLE by hyperactivating NK cells via the TLR2‐MyD88‐NF‐κB pathway, leading to amplified systemic inflammation and aggravated LN. These findings underscore the potential of targeting *SA*‐EVs for therapeutic intervention in SLE.

## Graphical abstract

In SLE patients, *Streptococcus anginosus* is highly abundant in the gut microbiota. The SA secrete the extracellular vesicles (EV) that contain lipoteichoic acid (LTA) and bind to toll‐like receptor 2 (TLR2) on NK cells, then NK cells are activated via the MyD88‐NF‐κB pathway to produce pro‐inflammatory cytokines. This activated NK cells, secreting TNF, IL‐6, IL‐17A and IL‐22 proinflammatory cytokines, are pernicious for human nephritic cells, which exacerbate the pathogenesis of SLE‐associated lupus nephritis (LN). Thus, *SA*‐EVs (*S. anginosus*‐derived extracellular vesicles) through the TLR2‐MyD88‐NF‐κB pathway activate NK cells to produce proinflammatory cytokines that exacerbate the pathogenesis of SLE‐associated LN.

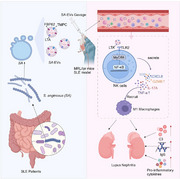

## 1 Introduction

Systemic lupus erythematosus (SLE) represents a paradigmatic autoimmune disorder that affects approximately 0.5% of the global population, with a markedly higher prevalence observed in females compared to males (Hoi et al. 2024; Siegel and Sammaritano 2024). Its global prevalence of SLE is estimated to range between roughly 20 and 150 per 100,000 across different regions. The clinical spectrum of SLE is extensive, encompassing a variety of manifestations such as cutaneous lesions, arthritis, nephritis and hematologic cytopenias. A defining feature of SLE is the presence of autoantibodies, particularly anti‐nuclear antibodies (ANA) and Anti‐double‐stranded DNA Antibodies (Anti‐dsDNA), which have the capacity to cross‐react with self‐antigens, thereby inciting inflammatory responses and resulting in tissue damage (Lazar and Kahlenberg 2023; Morand et al. 2023). Although the precise aetiology of SLE remains elusive, it is widely attributed to a complex interplay of genetic, environmental, hormonal and immune dysregulation factors (Tsokos 2011). Recently, research has increasingly explored the potential role of pathogenic infections in the development of SLE.

Emerging evidence indicates that environmental factors play a pivotal role in SLE pathogenesis (Jin et al. 2025). The human gut microbiome is crucial for immune homeostasis, and perturbations in gut microbial dysbiosis have been linked to immune dysregulation in autoimmune diseases including SLE (Silverman et al. 2024; Vieira et al. 2021). Indeed, metagenomic analyses reveal that SLE patients frequently exhibit a dysbiotic gut microbiota (Silverman et al. 2024), characterised by a depletion of non‐pathogenic bacteria, such as *Bifidobacterium*, alongside a concomitant expansion in opportunistic pathogens, notably *Streptococcus anginosus* and *Streptococcus intermedius* (Tomofuji et al. 2022). Consistent with these observations, our own 16S rRNA sequencing found a high prevalence of *S. anginosus* in faecal samples from SLE patients (Li et al. 2019), suggesting that such microbial alterations may be involved in the onset and progression of SLE. *S. anginosus* is a Gram‐positive, non‐spore‐forming, non‐motile bacterium commonly located in the oral cavity, nasopharynx, gastrointestinal tract and vagina (Pilarczyk‐Zurek et al. 2022). It is known to cause infections across various organs, including brain abscesses, liver abscesses, peritonsillar abscesses, chronic maxillary sinusitis, gastritis and endocarditis (Fu et al. 2024; Pilarczyk‐Zurek et al. 2022).

Nowadays, the extracellular vesicles (EVs)secreted from gut microbial have been recognised as important mediators of disease pathogenesis (Duan et al. 2025). Bacterial extracellular vesicles (BEVs), which consist of a lipid bilayer and range from 20 to 500 nm in diameter, are secreted by bacteria during growth (Wen et al. 2023). BEVs are ubiquitous among pathogenic microorganisms and encapsulate a variety of bacterial components, including nucleic acids, toxins, lipoproteins and enzymes (Parveen and Subramanian 2022). They function as vectors for bacterial communication by transporting molecular cargo, intercepting bacteriophages and antibiotics, and facilitating the transfer of virulence factors and resistance genes (Ou et al. 2023). Importantly, these vesicles not only enable bacterial communication but also modulate host cellular responses, thereby influencing immune reactions (Mehanny et al. 2020). Studies indicate that EVs can impact the host immune system by either promoting the release of inflammatory mediators or suppressing host defence mechanisms, thus allowing pathogens to evade immune clearance and proliferate (Liu et al. 2024; Wen et al. 2023). Some BEVs carry virulence factors, interact with host immune cells and induce intense immune responses that result in host tissue damage (Ou et al. 2023).

*S. anginosus*, in particular, harbours numerous virulence factors that facilitate its adhesion to host tissues and evasion of immune defences, including several surface‐associated proteins that bind to extracellular matrix components such as fibronectin, fibrinogen, collagen and laminin (Kuryłek et al. 2022). For instance, the gene *fbp62*, which encodes a fibronectin‐binding protein in *S. anginosus* (NCTC 10713), enables the bacterium to adhere to immobilised fibronectin and epithelial cells, while a mutant strain lacking this gene, Δ*fbp62*, exhibits a diminished capacity for adhesion (Kodama et al. 2018). Additionally, *S. anginosus* secretes hyaluronidase, which causes tissue liquefaction and pus formation, thereby increasing connective tissue permeability and facilitating the dissemination of bacteria and toxins. A recently identified antigen, *S. anginosus* antigen (*SA*A), can stimulate macrophages to produce nitric oxide (NO) and TNF‐α mRNA (Kumar et al. 2024). Furthermore, *S. anginosus* secretes additional virulence factors, including PsaA, PavB and Antigen I/I, which are believed to play roles in bacterial adhesion and tissue invasion, although their precise mechanisms remain to be fully elucidated (Kuryłek et al. 2022). Notably, the TMPC protein on the surface of *S. anginosus* interacts with the Annexin A2 (ANXA2) receptor on gastric epithelial cells, promoting bacterial adhesion, colonisation and activation of the mitogen‐activated protein kinase (MAPK) signalling pathway (Fu et al. 2024). Hence, it is conceivable that EVs shed by *S. anginosu*s carry similar virulence determinants or signals, thereby contributing to SLE pathogenesis through analogous mechanisms.

However, research on the specific impact of *S. anginosus*‐derived EVs on SLE pathogenesis remains largely unexplored. Therefore, the present study aims to elucidate the mechanisms by which *S. anginosus* EVs influence SLE, particularly their role in initiating the disease. A more comprehensive understanding of the interactions between *S. anginosus* EVs and the host in SLE could illuminate the disease's underlying aetiology and offer novel targets for its prevention and treatment.

## 2 Materials and Methods

### 2.1 Research Participants and Sample Collection

A total of 54 patients with SLE and 43 healthy controls (HC) were consecutively recruited from Nanfang Hospital, Southern Medical University in 2023. Each SLE patient met the American College of Rheumatology (ACR) classification criteria for SLE (Aringer and Johnson 2020; Aringer and Petri 2020; Li et al. 2019). Patients with acute intercurrent illnesses, infections or recent use of probiotics or antibiotics within one month prior to admission were excluded. Gender‐ and age‐matched HC participants with no history of autoimmune diseases were also recruited from the Health Examination Centre of Nanfang Hospital. Comprehensive participant information is presented in Tables 1 and S1. Fresh faecal samples from all participants were immediately frozen at ‐80°C following collection. SLE disease activity (SLEDA) was assessed using the SLEDA Index (SLEDAI), following previously described methods (Li et al. 2019; Morand et al. 2023). All the blood, serum and faecal samples were collected at the time of patient enrolment, prior to the initiation of any immunosuppressive therapy or antibiotic treatment. Additionally, disease activity was assessed using the SLEDAI at the time of sample collection. Ethics approval was granted by the Nanfang Hospital Ethics Committee, with all procedures conducted in accordance with approved guidelines (NFEC‐2025‐026). Informed written consent was obtained from all patients and healthy volunteers.

**TABLE 1:** Characteristics of the participants for blood, serum and faecal samples collection, related to Figure 1.

Characteristic	SLE	HC	*p* value (HC vs. RA)
Participants	**54**	**43**	/
AGE	41.33 ± 14.29	46.790 ± 13.17	0.561^a^
BMI	23.25 ± 2.14	22.83 ± 1.77	0.7736^a^
Gender	48F, 6M	39F, 4M	0.7476^b^
Duration (y)	7.82 ± 5.97	/	/
SLEDAI score	11.46 ± 4.82	/	/
Anti‐dsDNA antibody	44 positive	/	/
Anti‐ANA antibody	40 positive	/	/
Anti‐Sm antibody	35 positive	/	/

*Note*: Data were shown as Mean ± SD.

^a^ *p* value was calculated by Wilcox test.

^b^ *p* value was calculated by Chi‐squared test.

Venous blood specimens were collected in EDTA‐Na2 anticoagulant tubes, with serum harvested following 2000 ×*g* centrifugation for 10 min (Li et al. 2022). Faecal samples were transported in frozen, anaerobic conditions and stored long‐term at −80°C (Hong et al. 2023). All venous blood analyses were conducted within 24 h of specimen receipt.

### 2.2 Reagents

BD BACTO Brain Heart Infusion (BHI broth) (Cat: 237500) were purchased from BD Bioscience (New Jersey, USA). Anti‐Granzyme B‐PE (Cat: 372208), anti‐human‐TNF‐α‐BV605 (Cat: 502936, BioLegend) and anti‐human‐NKG2A—PE/Cyanine7 (Cat: 375113, BioLegend) were purchased from BioLegend (San Diego, USA). Gram‐positive bacteria LTA (lipoteichoic acid) monoclonal antibody (Cat: MA1‐7402), cell staining DIL dye (Cat: D282) and cell stimulation cocktail (Cat: 00‐4970‐03) were purchased from Thermo Fisher Scientific (Waltham, Massachusetts, USA). Human‐IL‐6 (Cat: E‐EL‐H6156), human‐IL‐17A (Cat: E‐EL‐H5812), human‐IL‐22 (Cat: E‐EL‐H0106), human‐TNF‐α(Cat: E‐EL‐H0109), human‐Granzyme B (Cat: E‐EL‐H1617), human‐MIP‐1α(Cat: E‐EL‐H6213), human‐MCP‐1 (Cat: E‐EL‐H6005), human‐CXCL8 (Cat: E‐EL‐H6008) and mouse‐MIP‐1α(Cat: E‐EL‐M0007) ELISA kit were purchased from Elabscience (Wuhan, China). BD Cytofix/Cytoperm Fixation/Permeabilisation Kit (Cat: 554714), anti‐human TIM3‐BV605 (Cat: 569245), anti‐human TIGIT‐BV421 (Cat: 747844), anti‐human CD69‐PEcy7 (Cat: 335792), anti‐human CD25‐PEcy7 (Cat: 557741), anti‐human CD56‐APC (Cat: 341025), anti‐human CD3‐BV510 (Cat: 564713) from BD Bioscience. Anti‐Human TLR2‐PE (392306), TLR4‐PE (312806), TLR6‐PE (334708), TLR7‐PE (376904) and TLR9‐PE (394804) were purchased from Biolegend. Western blot antibody anti‐TLR2 antibody (Cat: 12276T), phosphate‐P65 (Cat: 3033T), P65 (Cat: 8242T), IκBα (Cat: 4812T), phosphate‐ IκBα (Cat: 2859T), MyD88 (Cat:4283T), β‐Actin (Cat: 4967S) were bought from Cell Signalling Technology (Massachusetts, USA). Mouse‐IL‐6 (Cat: PI326), mouse‐IL‐17A (Cat: PI545), mouse‐IL‐22 (Cat: PI591), mouse‐TNF‐α (Cat: PT512) and mouse IFN‐β (Cat: PI568) ELISA kits were bought from Beyotime Biotechnology (Shanghai, China). Mouse Anti dsDNA Igs(Total A+G+M) (Cat: 5110) and Mouse Anti‐Nuclear Antigens (ANA/ENA) Ig's (total (A+G+M)) (Cat: 5210) ELISA Kits were bought from Alpha Diagnostic (San Antonio, USA). LTA ELISA kit (Cat: AKR‐5153) was bought from CELL BIOLABS (San Diego, USA). TLR2 inhibitor (C29, Cat: HY‐100461), TLR4 (Stepharine, HY‐N9347), TLR7 (Enpatoran, HY‐134581A), TLR9 (AT791, HY‐124603) and MyD88 inhibitor (MyD88‐IN‐1, Cat: HY‐149992) were obtained from MedChemExpress (MCE, USA). Anti‐mouse Nephrin (Cat: AF3159; R&D Systems, USA), Goat anti Mouse IgG (Cat: A10524, Thermo Fisher Scientific), C3 (Cat: NB200‐ 540, AF488; Novus, USA), F4/80 (Cat: 30325T, CST), CD206 (Cat: 24595T, CST), NKp44 (Cat: MA5‐44850, Thermo Fisher Scientific), NKp46 (Cat: PA5‐102860, Thermo Fisher Scientific), TNF‐α (Cat: 11948T, CST) and activated caspase‐3 (Cat: 9664T, CST) antibodies were used for immunofluorescence staining.

### 2.3 Cell Culture

RPTEC/TERT1 cells (Cat: CRL‐4031) and HK‐2 cells (Cat: CRL‐2190) were obtained from ATCC. RPTEC/TERT1 cells were cultured in DMEM:F12 Medium (Cat: 10565018, Thermo Fisher Scientific) supplemented with 10% FCS (Cat: A5256701, Thermo Fisher Scientific) and 1% Pen/Strep (Cat: 15070063, Thermo Fisher Scientific). HK‐2 cells were maintained in a Keratinocyte SFM Kit supplemented with 0.05 mg/mL BPE and 5 ng/mL EGF (Cat: 17005042, Thermo Fisher Scientific). All cell culture experiments were conducted in a humidified incubator at 37°C with 5% CO₂.

### 2.4 NK Cells MACS Isolation and Cytotoxicity Assay

Primary human NK cells were isolated from SLE patients and healthy donor buffy coats (Nanfang Hospital, Guangzhou, China) utilising a MACS‐based negative selection isolation kit (130‐092‐657, Miltenyi Biotec, Bergisch Gladbach, Germany), as previously described. NK cells were subsequently cultured in complete NK medium (Nobimpex, Taizhou, Jiangsu, China) enriched with IL‐2 (1,000 IU/mL; Proleukin, Clinigen, Yardley, PA) (Gong et al. 2024).

NK cell cytotoxicity against target cells was evaluated using a 4‐h flow cytometry‐based assay. Tumour cells were labelled with CellTracker CM‐Dil Dye (Thermo Fisher Scientific) and incubated overnight. NK cells were then co‐cultured with labelled tumour cells at varying effector‐to‐target ratios (0.5:1, 1:1, 2:1 or 4:1) in duplicate. Following 4 h of co‐incubation, cell viability was assessed using a Live/Dead Fixable V500‐Aqua Dead cell stain kit (Thermo Fisher Scientific) and analysed on a Fortessa flow cytometer (BD Biosciences) with FlowJo software v.10 (BD Biosciences). Specific cytotoxicity was calculated using the formula: (% dead target cells—% spontaneous target cell death)/(100%—% spontaneous target cell death) × 100. (Gong et al. 2020).

### 2.5 TBNK Lymphocyte Counts and Flow Cytometry

The Multitest TM 6‐colour TBNK cell staining kit (Cat: 662995, BD) was used to quantify lymphocyte subpopulations as previously described (Li et al. 2022). The percentages and absolute counts of lymphocyte subpopulations, including CD3⁺ T cells, CD4⁺ T cells, CD8⁺ T cells, B cells and NK cells, were analysed on a BD Canto II flow cytometer (FCM) following standard operating procedures. Data analysis was performed using BD FACSDiva software v8.02 (Becton, Dickinson and Company, USA).

### 2.6 Statistical Analysis

Statistical analyses were performed using GraphPad Prism 9 (Graphpad Software, San Diego, CA). Results of the data in each group are displayed as means ± standard deviations (mean ± SD). Specific statistical tests are annotated within the respective figure legends.

## 3 Results

### 3.1 *S. anginosus* Is Notably Enriched in SLE Patients and Positively Correlates With Disease Severity

Previous research by our group (Li et al. 2019; Zhang et al. 2019), utilising 16sRNA sequencing of SLE patient faeces, revealed an increased abundance of Streptococcus species, aligning closely with other studies and identifying high expression of *S. anginosus* in the faeces of SLE patients (Tomofuji et al. 2022). Additional studies report that *S. anginosus* can induce disease conditions such as gastric ulcer and atrophic gastritis (Fu et al. 2024); however, whether it accelerates SLE progression in the gut remains to be explored further.

**FIGURE 1:**
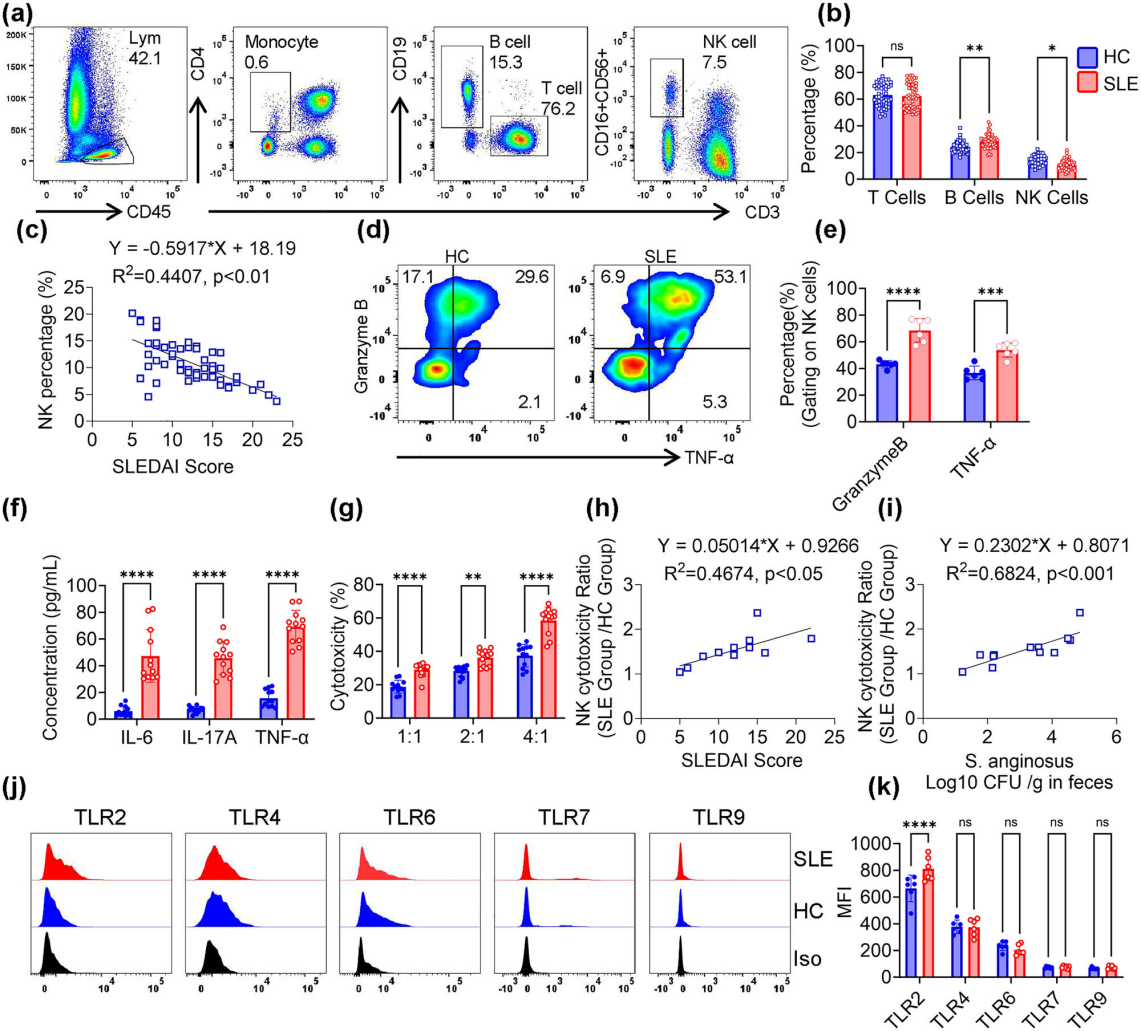
Reduced frequency yet heightened cytotoxicity of peripheral blood NK cells in SLE patients. (a) Representative flow cytometry plots depicting gating strategy for TBNK cell populations. (b) Quantitative analysis of TBNK subpopulations in SLE patients (*n* = 54) versus healthy controls (HCs; *n* = 43). (c) Correlation of peripheral blood NK cell percentage and SLEDAI score in SLE patients. (d) Intracellular expression of Granzyme B and TNF‐α in primary human NK cells following stimulation with PMA (10 ng/mL), ionomycin (1 µg/mL) and brefeldin A (BFA; 1 µg/mL) for 12 h, analysed by flow cytometry. (e) Quantification of granzyme B and TNF‐α expression. (f) Serum concentration of IL‐6, IL‐17A and TNF‐α in the serum of HC and SLE (*n* = 12). (g) The cytotoxicity of NK against K‐562 cells at varying effector: target cell ratios. NK cells were purified from PBMC. NK cells and target cells were co‐cultured with tumour cells for 4 h. (h) Correlation between NK cell percentage and SLEDAI score in SLE patients (*n* = 12). (i) Correlation of NK cell cytotoxicity with *Streptococcus anginosus* abundance in SLE patients (*n* = 12). (j) Surface expression of TLR2, TLR4, TLR6, TLR7 and TLR9 on human NK cells. (k) Quantitative analysis of TLR receptor mean fluorescence intensity (MFI) on NK cells (*n* = 6). Data are shown as mean ± SD, with individual data points representing biological replicates (average of technical duplicates). Statistical comparisons were performed using one‐way ANOVA with Tukey post‐tests. *p < 0.05; **p < 0.01; ***p < 0.001; ****p < 0.0001. ns indicates not significant.

To elucidate the inflammatory and gut microbiota signatures in SLE patients, we collected blood cells, serum and stool samples from SLE patients (*n* = 54) and HCs (*n* = 43, Tables 1 and S1). Using quantitative PCR, the absolute colony count of *S. anginosus* in SLE and HC faecal samples were detected (Figure S1a), which significantly higher colony counts in SLE patients' faeces samples were observed (Galazzo et al. 2020; Zhang et al. 2020). Moreover, the colony counts of *SA* positively correlated with SLEDAI (Figure S1b). Next, we evaluated if these gut microbiota alterations lead to systemic immune changes in SLE patients by analysing T‐, B‐ and NK‐cell subsets in peripheral blood of SLE patients and HCs (Figure 1a). In these comparisons, SLE patients exhibited elevated absolute and relative B cell counts, while NK cell counts were markedly reduced (Figures 1b, S2a and S3). Notably, both the percentages and absolute counts of NK cell were inversely correlated with disease severity (Figures 1c and S2a,b), and colony counts of *S. anginosus* were inversely correlated with NK cell counts (Figure S2c). Furthermore, NK cells from SLE patients showed increased secretion of granzyme and TNF‐α compared to NK cells from HCs (Figure 1d,e). Consistently, pro‐inflammatory cytokines (IL‐6, IL‐17A, TNF‐α) were also elevated in SLE patient serum compared to HCs (Figure 1f). We also observed increased NK‐cell cytotoxicity in SLE patients, which positively correlated with SLEDAI scores and with faecal *S. anginosus* counts (Figures 1g–I and S2d). Finally, the toll like receptor family on NK cells were stained with different antibodies and assessed by flow cytometry. Surprisingly, only TLR2 were significantly higher on NK cells from SLE patient than HC NK cells (Figure 1j,k).

### 3.2 *S. anginosus*‐Derived EVs Aggregate in Nephritic Tissue and Activate NK Cells to Produce Pro‐Inflammatory Cytokines

**FIGURE 2:**
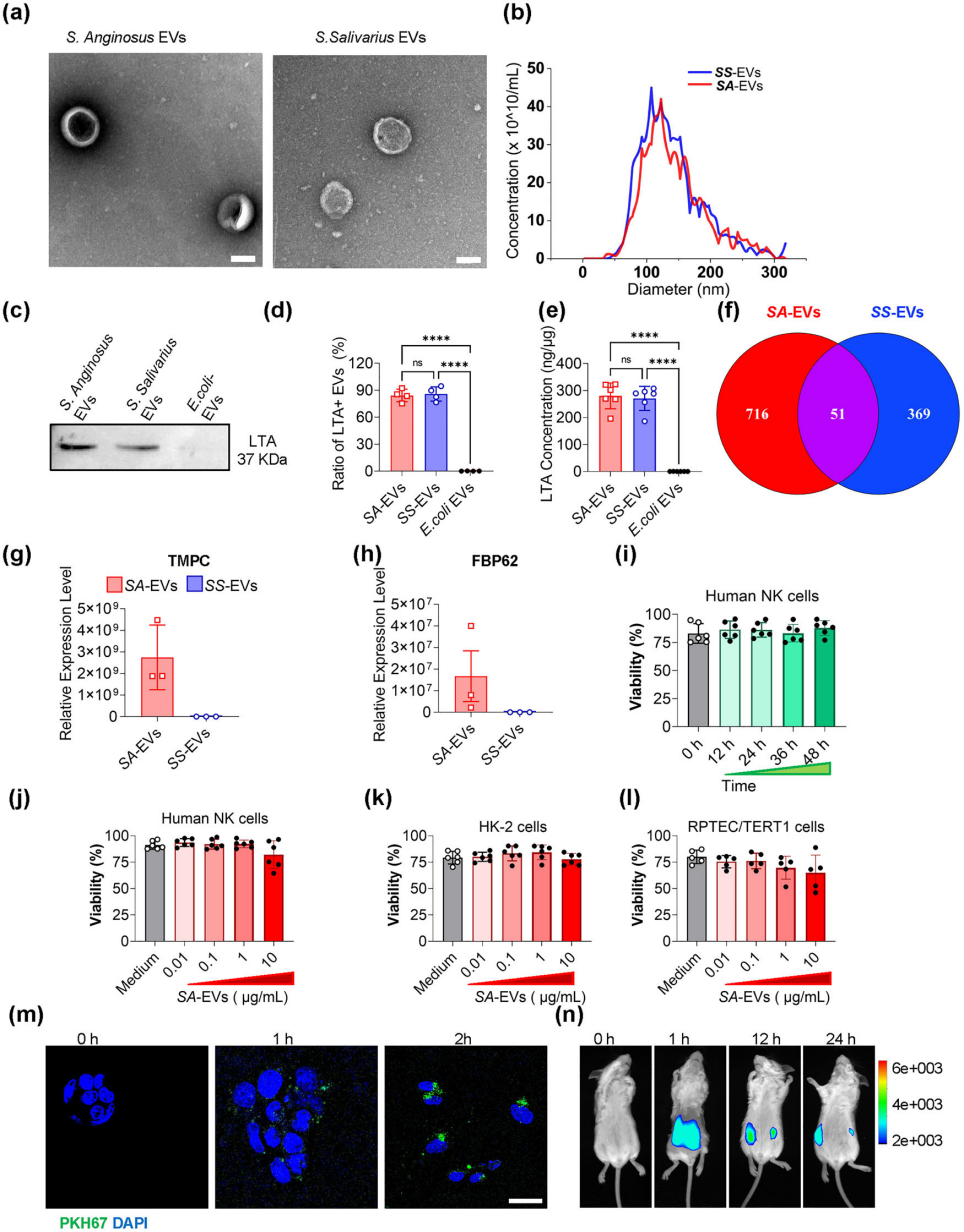
Characterisation of extracellular vesicles (EVs) derived from *Streptococcus anginosus* and their effects on human cells. (a) Transmission electron microscope (TEM) images of *SA‐*EVs. Scale bar: 100 nm. (b) Size distribution analysis of *SA‐*EVs by nanoparticle tracking analysis (NTA). (c) Western blot detection of lipoteichoic acid (LTA) in *S. anginosus, SA‐*EVs and *E.coli* EVs. (d) Quantitative densitometric analysis of LTA bands in SA‐EVs and E. coli EVs (ImageJ). (e) LTA concentration in *SA‐*EVs measured by ELISA. (f) Venn diagram illustrating peptide expression overlap between *SA‐*EVs and *S. salivarius (SS*‐EVs) as determined by DIA‐MS. (g, h) Relative expression of virulence factors TMPC and FBP62 in *SA‐*EVs versus *SS*‐EVs.(i) Time course analysis of the viability of NK cells following exposure to 1 µg/mL *SA‐*EVs. (j–l) Viability of NK cells, HK‐2 cells and RPTEC/TERT1 renal epithelial cells treated with varying concentrations of *SA‐*EVs. (m) Uptake of PKH67‐labelled *SA‐*EVs RPTEC/TERT1 cells. Scale bars, 50 µm. (n) Biodistribution of deep red‐labelled *SA‐*EVs in MRL/lpr mice following oral gavage, assessed by in vivo imaging. Data are presented as mean ± SD, with individual data points representing biological replicates (average of technical duplicates). Statistical comparisons were performed using one‐way ANOVA with Tukey post‐tests. *p < 0.05, **p < 0.01, ***p < 0.001, ****p < 0.0001. ns indicates not significant.

Due to the integrity of the mucosal immune barrier, *S. anginosus* rarely translocates into the bloodstream; however, its EVs can readily circulate and have been proposed as potential biomarkers for the infectious disease (Ou et al. 2023). Previous studies suggest that *Fusobacterium nucleatum* exacerbates rheumatoid arthritis by releasing FadA‐positive bacterial EVs, highlighting the role of circulating bacterial EVs in mediating interactions between the gut microbiota and the host immune system (Hong et al. 2023). To investigate the contribution of *S. anginosus* in SLE, EVs from *S. anginosus* (*SA*‐EVs) and from *S. salivarius* (*SS*‐EVs) as a comparative control were purified (Figure 2a). The *SA*‐EVs, approximately 100 nm in diameter (Figure 2b), were enriched in LTA molecules (Figure 2c–e). Moreover, the peptide confluence is different from *SA*‐EVs and *SS*‐EVs (Figure 2f–h). Importantly, the *SA*‐EVs neither induce NK cells nor renal tubular epithelial cell (HK‐2, RPTEC/TERT1) apoptosis (Figure 2i–l). *SA*‐EVs were efficiently internalised by cells within 2 h (Figure 2m) and, following oral administration to mice (20 µg), they preferentially accumulated in renal tissue, persisting for more than 24 h (Figure 2n).

**FIGURE 3:**
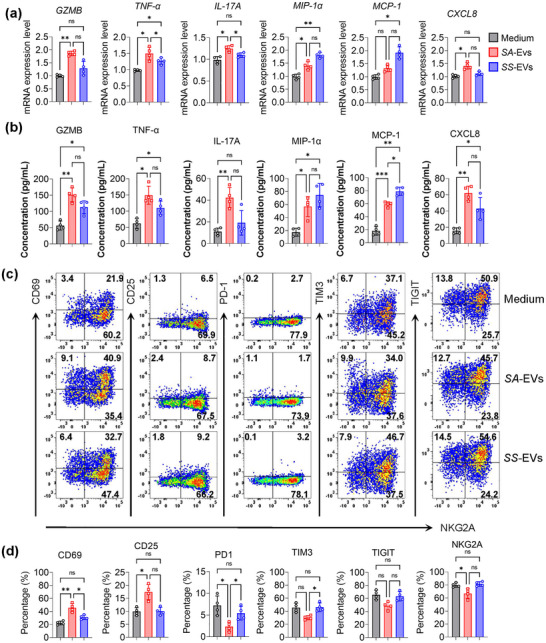
*SA‐*EVs activate human primary NK cells to produce proinflammatory cytokines. (a) RT‐ qPCR analysis of granzyme B, TNF‐α, IL‐17, MIP‐1α, MCP‐1 and CXCL8 mRNA expression in human primary NK cells after 24 h exposure to *SA‐*EVs (1 µg/mL) (*n* = 4). (b) ELISA quantification of granzyme B, TNF‐α, IL‐17, MIP‐1α, MCP‐1 and CXCL8 cytokines secreted by human primary NK cells following 48‐h exposure to *SA‐*EVs (1 µg/mL) (*n* = 4). (c) Representative flow cytometry plots depicting expression of activation (CD69, CD25) and inhibitory (NKG2A, TIM‐3, TIGIT) markers on NK cells after 48‐h incubation with *SA‐*EVs(1 µg/mL). (d) Quantitative analysis of activation and inhibitory marker expression in human primary NK cells treated with 1 µg/mL *SA‐*EVs for 48 h (*n* = 4). Data are presented as mean ± SD, with individual data points representing biological replicates (average of technical duplicates). Statistical differences between groups were determined using one‐way ANOVA with Tukey post‐tests.*p < 0.05, **p < 0.01, ***p < 0.001, ****p < 0.0001. ns indicates not significant.

In vitro co‐culture of *SA*‐EVs with human primary NK cells resulted in pronounced upregulation of pro‐inflammatory mRNA and proteins, including granzyme B, TNF‐α, IL‐17, MIP‐1α, MCP‐1 and CXCL8 (Figures 3a,b and S3). Moreover, *SA*‐EVs enhanced NK cell activation by increasing the expression of surface markers CD25 and CD69, while concurrently downregulating inhibitory receptors such as PD‐1, NKG2A, TIM‐3 and TIGIT (Figures 3c,d, S3 and S4). Collectively, these findings demonstrate that, compared to *SS*‐EVs, *SA*‐EVs more robustly activate NK cells, driving them toward a pro‐inflammatory phenotype.

### 3.3 *SA*‐EVs Activate Human NK Cells via the TLR2–MyD88–NF‐κB Signalling Pathway

**FIGURE 4:**
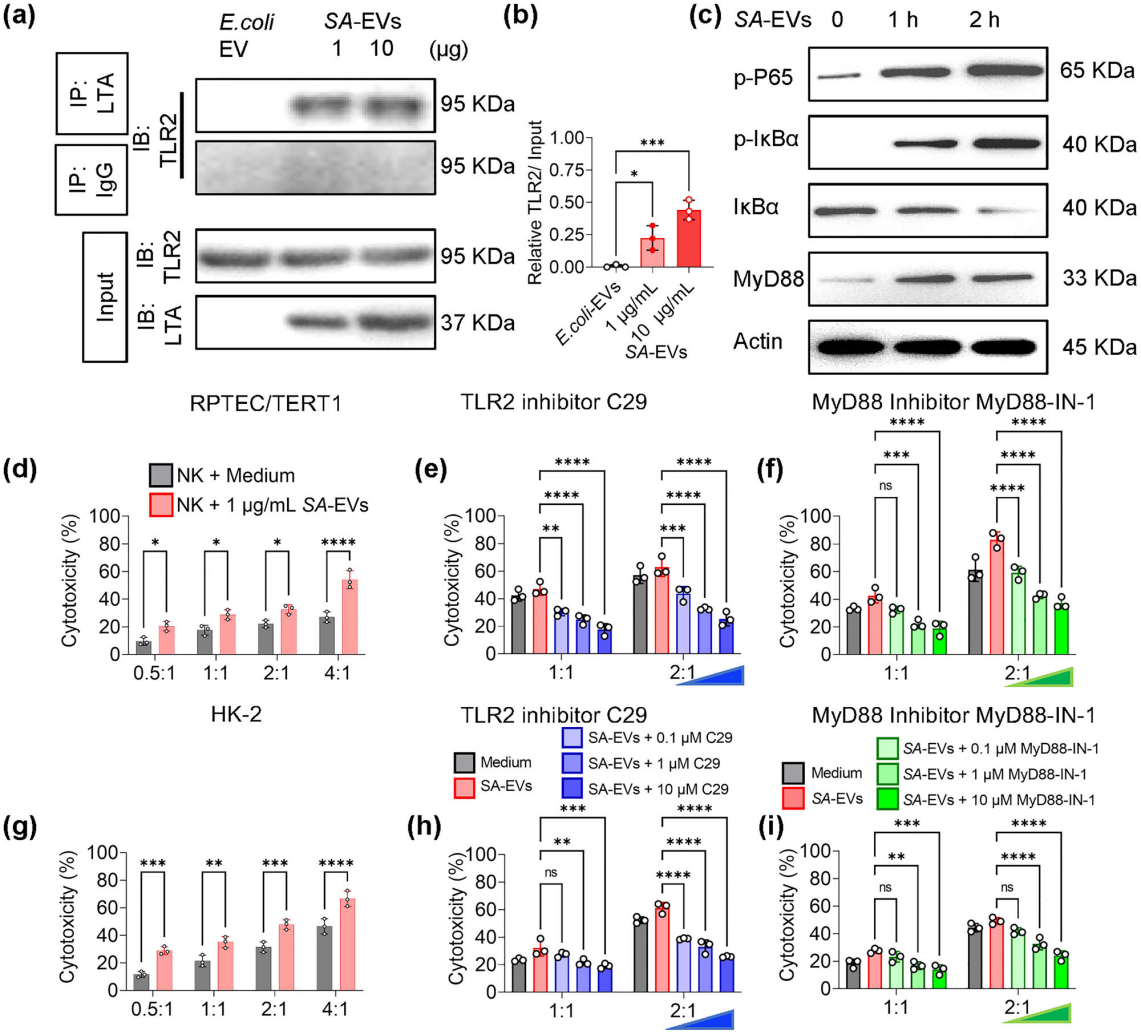
TLR2‐MyD88‐NF‐κB pathway mediates *SA‐*EVs‐induced activation of NK cells. (a) Immunoprecipitation (IP) analysis demonstrating interaction between LTA and TLR2 (*n* = 3). (b) Quantification of IP assay results. (c) Western Blot assay of NK cells following coculture with *SA‐*EVs(1 µg/mL), phosphorylation of P65, IκBα and MyD88 was measured (*N* = 3). (d and g) The cytotoxicity of NK cells against RPTEC/TERT1 (d) and HK‐2 (g) cells with or without 1 µg/mL *SA‐*EVs coculture in different effector: target (E:T) ratios. Target cells were labelled with Dil membrane dye, and dead cells were identified by DAPI staining. All the cytotoxicity assay data were acquired by flow cytometry. NK cells were preincubated with 1 µg/mL *SA‐*EVs coculture for 24 h (*N* = 3). (e and h) NK cell cytotoxicity against RPTEC/TERT1 (e) and HK‐2 (h) cells in the presence of the TLR2 inhibitor C29, across varying E:T ratios (*n* = 3). (f, i) NK cell cytotoxicity against RPTEC/TERT1 (f) and HK‐2 (i) cells in the presence of the MyD88 inhibitor MyD88‐IN‐1, across varying E:T ratios (*n* = 3). Data are presented as mean ± SD, with individual data points representing biological replicates (average of technical duplicates). Statistical analysis was performed using one‐way ANOVA with Tukey post‐tests. *p < 0.05, **p < 0.01, ***p < 0.001, ****p < 0.0001. ns indicates not significant.

Previous studies have demonstrated that LTA from *Staphylococcus aureus* can stimulate NK cell activation TLR2 engagement (Lauzon et al. 2006; Morath et al. 2002). To delineate the mechanism by which *SA‐*EVs induce pro‐inflammatory cytokine release in NK cells, co‐culture experiments with *SA‐*EVs and NK cells were conducted. Immunoprecipitation assays confirmed that LTA on *SA‐*EVs binds to TLR2 on NK cells (Figure 4a,b). Western blot analysis further revealed that *SA‐*EVs‐treated NK cells exhibited upregulation of MyD88 and activation of NF‐κB signalling components, specifically phosphorylated p65 and IκBα (Figure 4c), suggesting that *SA‐*EVs activate NK cells through the TLR2‐MyD88‐NF‐κB signalling axis (Figure 4c). In subsequent co‐culture assays of NK cell and renal tubular epithelial cell (RPTEC/TERT1 and HK‐2), *SA‐*EVs‐activated NK cells showed significantly heightened cytotoxicity against target cells (Figure 4d,g). Additionally, *SA‐*EVs promoted the secretion of TNF‐α and granzyme B from NK cells (Figure S5a,b). Notably, these pro‐inflammatory effects were abrogated by TLR2 inhibitors (C29) (Figure 4e,h) and MyD88 inhibitors (MyD88‐IN‐1) (Figure 4f,i), but remained unaffected by inhibitors of TLR4, TLR7 and TLR9 (Figure S6a–c). These findings provide further compelling evidence that *SA*‐EVs mediate NK cell activation predominantly through the TLR2‐MyD88‐NF‐κB signalling pathway.

### 3.4 *SA*‐EVs Exacerbate SLE Disease Severity by Activating Nephritic NK Cells

**FIGURE 5:**
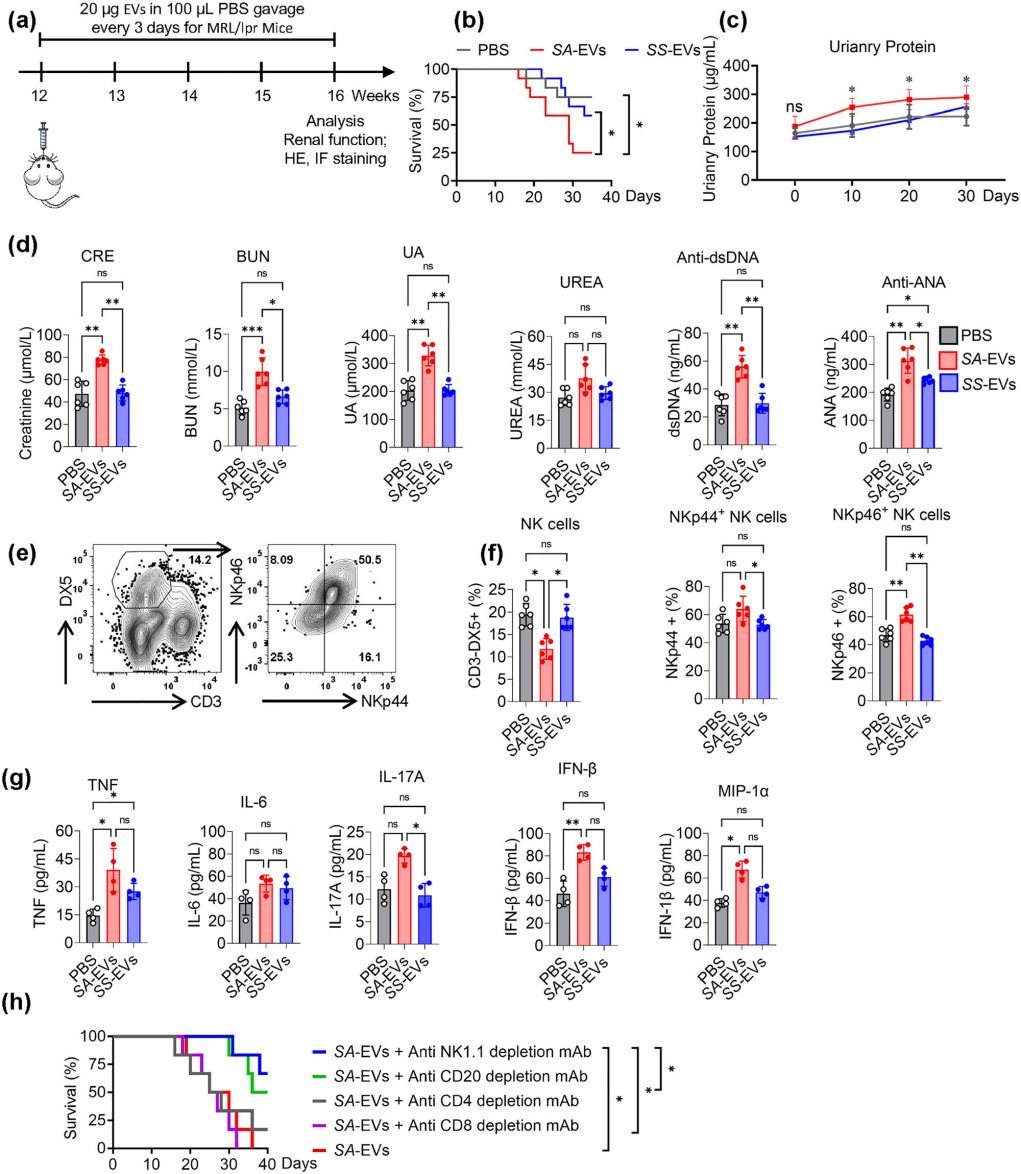
*SA‐*EVs exacerbate SLE disease severity via activation of nephritic NK cells. (a) Experimental schematic of SA‐EV oral gavage in MRL/lpr mice. Mice were received 100 µL PBS, 20 µg *SS*‐EVs or 20 µg *SA‐*EVs in 100 µL, administered every 3 days for 4 weeks. (b) Kaplan‐Meier survival curves of MRL/lpr mice following different EV treatments. (c) Time‐course analysis of urinary protein level in MRL/lpr mice subjected to different treatments. (d) Serum levels of creatinine (CRE), blood urea nitrogen (BUN), uric acid (UA), urea (UREA), anti‐dsDNA antibody (Anti‐dsDNA) and anti‐ANA antibodies (Anti‐ANA) after 4 weeks of treatment. (e) Flow cytometry analysis of nephritic infiltration NK cells; representative plots of NK cell markers NKp44 and NKp46. (f) Quantification of NK cells percentage, NKp44^+^ and NKp46^+^ cells among nephritic infiltration lymphocytes (*n* = 6). (g) Serum concentrations of proinflammatory cytokines TNF, IL‐6, IL‐17A, IFN‐1β and MIP‐1α (*n* = 4). (h) Kaplan‐Meier survival curves of MRL/lpr mice following specific immune cell depletions. Data is shown as mean ± SD, with dots representing individual donors (average of technical duplicates). Statistical differences between groups were determined using one‐way ANOVA with Tukey post‐tests.*p < 0.05, **p < 0.01, ***p < 0.001, ****p < 0.0001. ns indicates not significant.

To investigate whether *SA‐*EVs influence SLE progression in vivo, we orally administered *SA*‐EVs to MRL/lpr mice, a well‐established murine model of lupus. Within 1 h post‐gavage, *SA‐*EVs were detectable in both the liver and kidneys, with peak accumulation in the kidneys observed at 12 h (Figures 2n and S7). Following 4 weeks of *SA*‐EVs treatment (Figure 5a), survival rates in the *SA‐*EVs‐treated MRL/lpr mice were significantly reduced compared to both PBS‐treated controls and the *SS*‐EVs‐treated group (Figure 5b). Although *SA*‐EVs were capable of transferring specific DNA fragments to the host, they did not affect overall body weight (Figure S8a). In contrast, *SA*‐EVs administration significantly aggravated renal injury, as evidenced by increased proteinuria (Figure 5c), elevated serum levels of creatinine, urea nitrogen and uric acid, and heightened titres of anti‐dsDNA and ANA (Figure 5d).

To determine whether *SA‐*EVs promote NK cell activation in the kidneys, we isolated and analysed renal infiltrating lymphocytes (Figure 5e). Although the proportion of renal NK cells (CD3^−^DX5^+^) was lower in *SA*‐EVs‐treated mice compared to PBS controls (Figure 5f), these NK cells exhibited markedly increased expression of activation markers NKp44 and NKp46 (Figure 5f). Furthermore, ELISA analysis of serum from *SA‐*EVs‐treated mice revealed significantly elevated levels of pro‐inflammatory cytokines, including TNF‐α, IL‐6, IL‐17A, IFN‐1β and MIP‐1α (Figure 5g).

Finally, to identify the immune cell subsets contributing most prominently to *SA*‐EVs‐induced mortality, we conducted antibody‐mediated depletion of specific lymphocyte populations. Interestingly, depletion of NK cells (anti‐NK1.1) and B cells (anti‐CD20) both prolonged survival, with NK‐cell depletion conferring a more pronounced survival benefit compared to B‐cell depletion following 40 days of *SA*‐EVs treatment (Figure 5h).

### 3.5 *SA*‐EVs Aggravate Pathological Alterations in Lupus Nephritis (LN) in an Experimental SLE Mouse Model

**FIGURE 6:**
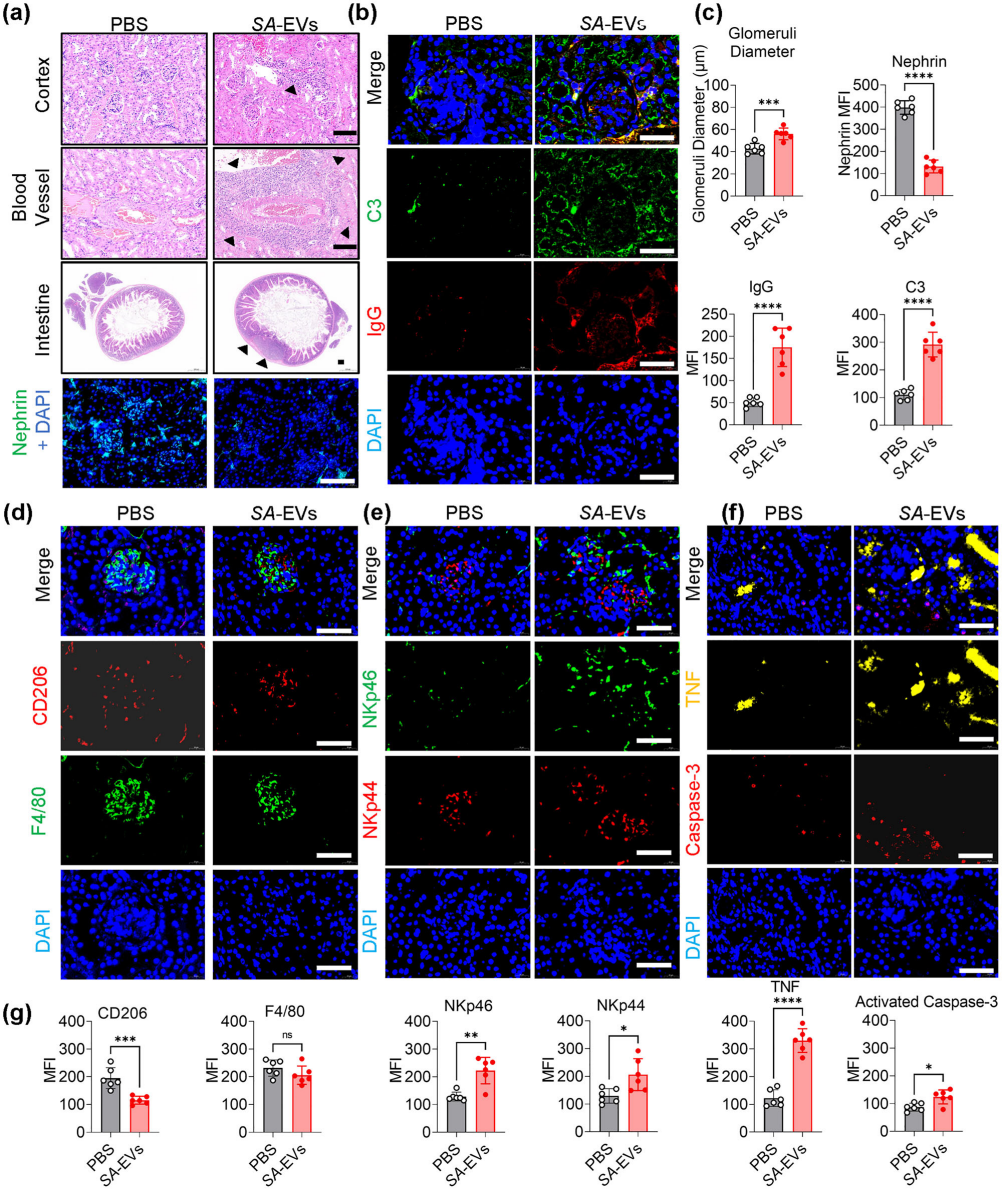
*SA‐*EVs exacerbate lupus nephritis histopathological changes in an experimental SLE mouse model. (a) Representative histopathological images of kidney cortex, blood vessels, intestinal lymph nodes and glomerular nephrin expression in MRL/lpr mice. Haematoxylin and eosin (H&E) staining; scale bar = 50 µm. Black triangles indicate infiltrating inflammatory immune cells. (b) immunofluorescence images showing glomerular deposition of IgG and complement C3 in kidneys of MRL/lpr mice; scale bars = 50 µm. (c) Quantitative analysis of the glomerular size, the expression of nephrin and the mean fluorescence intensity of IgG and C3. (d) Expression of CD206 and F4/80 markers in the glomeruli of the kidneys from MRL/lpr lupus mice. Scale bars = 50 µm. (e) Expression of NK cell activation markers NKp44 and NKp46 in the kidneys from MRL/lpr lupus mice. Scale bars = 50 µm. (f) Expression of TNF and activated caspase‐3 in the kidneys from MRL/lpr lupus mice. Scale bars = 50 µm. (g) Quantitative analysis of the expression of CD206, F4/80, NKp46, NKp44, TNF and activated Caspase‐3. Data is shown as mean ± SD, with dots representing individual donors (average of technical duplicates). Statistical differences between groups were determined using one‐way ANOVA with Tukey post‐tests. *p < 0.05, **p < 0.01, ***p < 0.001, ****p < 0.0001. ns indicates not significant.

Histological analysis of MRL/lpr mouse kidneys revealed *SA*‐EVs treatment significantly exacerbated renal pathology, characterised by extensive immune cell infiltration in the renal medulla, enlarged glomeruli (Figure 6a) and pronounced perivascular inflammatory infiltrates, compared to PBS‐treated controls (Figure 6a). Importantly, we detected *S. anginosus*‐specific DNA fragments in the renal tissues of mice following oral administration of *SA*‐EVs, using a probe specifically targeting *S. anginosus*. This finding indicates that *SA*‐EVs can localise and persist within renal tissues after gavage, providing further evidence that *SA*‐EVs accumulate in the kidneys (Figure S9). Increased lymphocyte and immune cell infiltration were also observed in visceral organs (Figure S8b), accompanied by downregulation of nephrin expression, indicative of glomerular injury (Figure 6a,c). Notably, *SA*‐EVs‐treated kidneys exhibited increased deposition of complement C3 and IgG immune complexes within glomeruli (Figure 6b,c). Analysis of macrophage polarisation revealed a shift towards a pro‐inflammatory M1 phenotype, as evidenced by decreased CD206 expression, while F4/80 levels remained unchanged (Figure 6d,g), suggesting that *SA*‐EV treatment amplified renal inflammation via macrophage reprogramming. Immunofluorescence staining further demonstrated that renal tissues from *SA‐*EV‐treated MRL/lpr mice displayed elevated expression of NK cell activation markers NKp44 and NKp46 localised around glomeruli (Figure 6e,g). Additionally, we observed increased expression of TNF‐α and activated caspase‐3, indicating enhanced inflammatory signalling and elevated levels of renal apoptosis (Figure 6f,g).

## 4 Discussion

This study provides novel insights into the complex interactions between the gut microbiota, specifically *S. anginosus* and systemic immune responses in patients with SLE. Our findings suggest that *SA*‐EVs engaged pattern‐recognition receptors on innate effector cells, notably Toll‐like receptor 2 (TLR2), initiating the canonical MyD88‐dependent NF‐κB signalling cascade. This activation led to marked production of proinflammatory cytokines and chemokines. Strikingly, exposure to *SA*‐EVs elicited robust activation of natural killer (NK) cells, as evidenced by upregulation of activation markers and enhanced effector cytokine secretion. These findings unveil a novel microbial mechanism for triggering innate lymphocyte activation and inflammatory signalling, which may contribute to the immunopathology of SLE.

Gut microbiota has been increasingly recognised for its role in modulating immune responses and maintaining systemic immune homeostasis (Wang et al. 2022; Zhu et al. 2024). Dysbiosis, or microbial imbalance, is frequently observed in autoimmune diseases, including SLE, where it may contribute to immune dysregulation and exacerbate disease symptoms (Li et al. 2023). In line with this, our study observed a significant alteration in the gut microbial composition of SLE patients, particularly an increased prevalence of *S. anginosus*. This dysbiotic environment likely creates conditions that impair the gut mucosal barrier, allowing microbial metabolites and bacterial components to interact with the host immune system more directly and potentially trigger systemic autoimmune responses (Jia et al. 2023; Pan et al. 2021). Such an environment might set the stage for *S. anginosus* and other pathobionts to interact with host immune cells, contributing to an inflammatory milieu that exacerbates SLE symptoms.

Our findings are significant as they add to the growing evidence of gut microbiota's potential role in influencing autoimmune disease severity and progression. *S. anginosus* is a facultative anaerobic bacterium commonly found in the oral cavity, gastrointestinal tract and genitourinary tract (Kodama et al. 2018; Kumar et al. 2024). Known for its role in abscess formation and tissue invasion, *S. anginosus* is emerging as a potential contributor to autoimmune conditions through its interactions with host immune cells (Kumar et al. 2024). Our study confirms that *S. anginosus* is not only enriched in the faeces of SLE patients but is also positively correlated with disease severity, as measured by the SLEDAI score diagram. This observation points to a possible relationship between *S. anginosus* levels and the clinical course of SLE, suggesting that the presence of this bacterium could act as a biomarker for disease progression and potentially as a therapeutic target.

A unique feature of *S. anginosus* is its ability to produce virulence factors, such as fibronectin‐binding proteins, which enhance its adhesion to host tissues and immune cells (Kuryłek et al. 2022). In the context of SLE, our study demonstrated that *S. anginosus* might contribute to autoimmune reactions by releasing EVs containing virulence factors (Kuryłek et al. 2022; Kumar et al. 2024). Notably, this interaction was found to activate NK cells via the TLR2‐MyD88‐NF‐κB pathway, promoting the release of pro‐inflammatory cytokines that are closely associated with SLE pathology. On one hand, the focus on a single pathobiont allows for a more detailed examination of its specific role in SLE, which adds clarity and depth to our understanding of microbial influences on autoimmune diseases. On the other hand, focusing solely on *S. anginosus* does not account for the interactions between multiple species within the gut microbiota. SLE is likely influenced by a complex microbial ecosystem, and while *S. anginosus* may play a key role, other microbial species and their interactions could also contribute significantly to disease mechanisms.

Our study also underscores the role of BEVs in SLE (Guo et al. 2020). BEVs are nano‐sized vesicles released by bacteria that carry a variety of bacterial components, including proteins, lipids and genetic material (Toyofuku et al. 2019). These vesicles serve as a means of communication between bacteria and the host and can modulate host immune responses (Aringer 2020; Kaparakis‐Liaskos and Ferrero 2015). EVs from *S. anginosus* were found to engage with NK cells, which are integral to the innate immune response. The activation of NK cells by BEVs led to increased secretion of pro‐inflammatory cytokines, including TNF‐α, IL‐17 and Granzyme B (Baschuk et al. 2014; Bluman et al. 1996; Montaldo et a 2012). This heightened inflammatory response in NK cells could exacerbate autoimmune processes in SLE, especially in tissues where these cells accumulate, such as the kidneys in LN.

The investigation of *SA‐*EVs offers significant advantages for understanding SLE pathogenesis. Unlike intact bacteria, *SA‐*EVs can pass through biological barriers and deliver their cargo directly into host cells, providing a unique means of triggering localised and systemic immune responses. This characteristic makes *SA‐*EVs particularly relevant in diseases like SLE, where systemic immune activation is a hallmark. However, one limitation of this study is that the exact components within *SA‐*EVs responsible for NK cell activation were not fully identified. Although we observed increased levels of LTA within *SA‐*EVs, it remains unclear whether LTA alone accounts for the observed immune activation or if other bacterial factors are also involved. Future studies should focus on characterising the molecular composition of *SA‐*EVs to identify specific components that trigger immune responses in SLE.

NK cells are known for their cytotoxic abilities and their role in recognising and eliminating infected or transformed cells (Vivier et al. 2024). However, in autoimmune diseases like SLE, NK cell function is often dysregulated (Tsokos 2024). Our study observed a significant reduction in NK cell counts in SLE patients’ peripheral blood, coupled with increased production of pro‐inflammatory cytokines by NK cells exposed to *SA‐*EVs. This paradox suggests that while the overall number of NK cells is reduced, those present are more prone to an inflammatory state, likely contributing to SLEDA. The NK cells in the context of SLE have their dual role in cytotoxicity and immune regulation. By focusing on NK cell activation in response to *SA‐*EVs, our research provides a more nuanced understanding of how NK cells can shift from protective to pathogenic roles under specific conditions (Tsokos 2024). The heightened inflammatory state of NK cells, particularly in renal tissues, likely exacerbates LN, a severe manifestation of SLE characterised by immune cell infiltration and tissue damage in the kidneys (Aringer 2020; Tsokos 2024). However, one limitation of this study is that it primarily focuses on the activation of NK cells and does not fully explore other immune cells that might also be affected by *SA‐*EVs. Given the complex immune dysregulation in SLE, it would be beneficial to investigate the interplay between NK cells and other immune cells, such as T and B cells, to obtain a more comprehensive view of immune activation in response to gut microbiota‐derived vesicles.

This study provides several important contributions to our understanding of the interactions between gut microbiota and immune responses in SLE. First, by focusing on *S. anginosus* and its *SA‐*EVs, we were able to delineate specific mechanisms through which gut‐derived bacterial factors may influence SLE pathology (Tsokos 2024). The study's use of both in vitro and in vivo models allowed us to observe immune activation in a controlled environment while also validating these findings in a murine model of SLE (Toyofuku et al. 2019). This dual approach strengthens the relevance of our findings and suggests that BEVs may indeed play a significant role in SLE pathogenesis. Additionally, our exploration of the TLR2‐MyD88‐NF‐κB pathway offers a potential target for therapeutic interventions that could dampen inflammatory responses associated with SLE (Aringer 2020; Dutta et al. 2021; Gallardo‐Zapata et al. 2024).

Our current data demonstrated that EVs from gut microbes carry microbial‐associated molecular patterns that activate host innate immunity and inflammation. By analogy, it will be important to test whether EVs from *S. anginosus* (and other gut bacteria) likewise induce immune cell activation. In light of this study, further studies are warranted to determine if EVs from additional pathogenic species can elicit similar inflammatory responses. Despite these advantages, several limitations should be acknowledged. One major limitation is the study's reliance on correlation‐based findings between *S. anginosus* levels and SLEDA, which does not definitively establish causation. Although our findings indicate a positive association between *S. anginosus* abundance and SLE severity, additional mechanistic studies, such as those using bacterial depletion or modification of microbiota composition, would be required to confirm a direct causal role. Moreover, our study predominantly focused on NK cell responses, while other immune cells, such as T and B cells, also play significant roles in SLE and may interact with bacterial EVs differently. A comprehensive analysis that includes these cell types would provide a fuller understanding of immune dysregulation in response to gut‐derived bacterial factors. Additionally, the use of a murine model of SLE to investigate the in vivo impact of *SA‐*EVs provides valuable insights but may not fully translate to human SLE pathology. Differences in immune system responses between mice and humans can limit the applicability of these findings to clinical settings. Further studies using humanised models or clinical samples could help bridge this gap and increase the translational potential of our research. Future studies should aim to isolate and characterise these components to better understand their immunostimulatory effects, which could lead to targeted therapeutic approaches to mitigate SLE progression by neutralising specific bacterial factors.

## 5 Conclusions

In conclusion, this study identifies a previously underappreciated pathogenic axis in lupus: the activation of innate immunity by *S. anginosus*–derived EVs. We demonstrate that *SA*‐EVs act as potent immunostimulatory agents, engaging the TLR2‐MyD88‐NF‐κB pathway to activate NK cells and propagate inflammatory cytokine networks. These findings expand current paradigms of SLE aetiology by implicating bacterial vesicles, not solely live microbes, as drivers of autoimmune inflammation. Clinically, our results suggest that targeting *SA*‐EVs or their TLR2‐activating components (for example, using TLR2 antagonists or inhibitors of vesicle biogenesis) could attenuate autoimmune pathology. Future studies should aim to characterise the specific molecular constituents of *SA*‐EVs responsible for TLR2 activation and to evaluate the therapeutic efficacy of disrupting this axis in preclinical models of LN. These findings establish a mechanistic link between gut dysbiosis, BEVs and autoimmune progression in SLE. Targeting *SA*‐EVs or their associated signalling pathways may represent a promising therapeutic approach for mitigating disease severity and improving clinical outcomes in SLE patients. Future investigations should further delineate the molecular components of *SA*‐EVs responsible for NK cell activation and explore potential interventions to modulate their effects in autoimmune diseases.

## Author Contributions

**Ying Gong**: conceptualization (supporting), data curation (equal), formal analysis (equal), funding acquisition (equal), investigation (equal), methodology (equal), resources (equal), software (equal), validation (equal), visualization (equal), writing–original draft (equal), writing–review and editing (equal). **Lingyue Jin**: Data curation (equal), formal analysis (equal). **Lina Duan**: Data curation (equal), formal analysis (equal). **Jie Xiao**: Data curation (equal), formal analysis (equal), software (equal), validation (equal), visualization (equal), writing–original draft (equal). **Yao Li**: Data curation (supporting). **Hongxia Wang**: Data curation (supporting), methodology (supporting), writing–original draft (supporting). **Haifang Wang**: Data curation (supporting), writing–original draft (supporting). **Wanying Lin**: data curation (supporting), funding acquisition (supporting) writing–original draft (supporting). **Yi Zhang**: Data curation (supporting), writing–original draft (supporting). **Xiufeng Gan**: Data curation (supporting), writing–original draft (supporting). **Shuyin Pang**: data curation (supporting), writing–original draft (supporting). **Yurong Qiu**: Conceptualization (supporting), writing–original draft (supporting). **Weinan Lai**: Conceptualization (equal), resources (equal), writing–original draft (equal), writing–review and editing (equal). **Lei Zheng**: Conceptualization (equal), funding acquisition (equal), project administration (equal), writing–original draft (equal), writing–review and editing (equal). **Haixia Li**: conceptualization (lead), funding acquisition (lead), investigation (equal), methodology (equal), project administration (lead), writing–original draft (equal), writing–review and editing (equal).

## Disclosure

Patients and/or the public were not involved in the design, conduct, reporting or dissemination plans of this research.

## Ethics Statement

This study involves human participants and was approved by the Ethics Committee of Nanfang Hospital, Southern Medical University (NFEC‐2025‐026). Participants gave informed consent to participate in the study before taking part.

## Conflicts of Interest

The authors declare no conflicts of interest.

## Supporting information




**Supporting Table 1**: jev270134‐sup‐0001‐tableS1.xlsx

**Supporting Fig. 1**: jev270134‐sup‐0002‐figuresS1‐S9.pdf

**Supporting Material**: jev270134‐sup‐0003‐SuppMat.docx

**Supporting Material**: jev270134‐sup‐0004‐SuppMat.xlsx

## Data Availability

Data are available on reasonable request.
