# Supplementary figures and images for: Extracellular Vesicles Derived From Streptococcus anginosus Aggravate Lupus Nephritis by Triggering TLR2‐MyD88‐NF‐κB Signalling in NK Cells
Source: J Extracell Vesicles. 2025 Jul 17;14(7):e70134. doi: 10.1002/jev2.70134 (PMC12269530; doi:10.1002/jev2.70134)

Figure S1

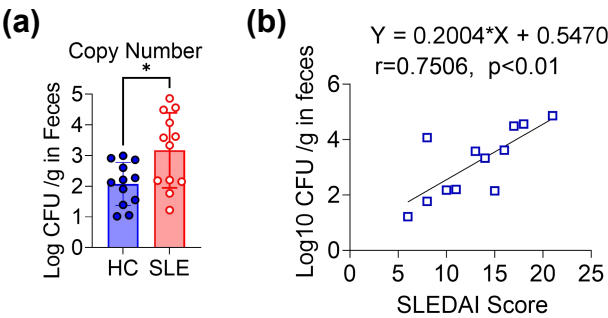

Figure S2

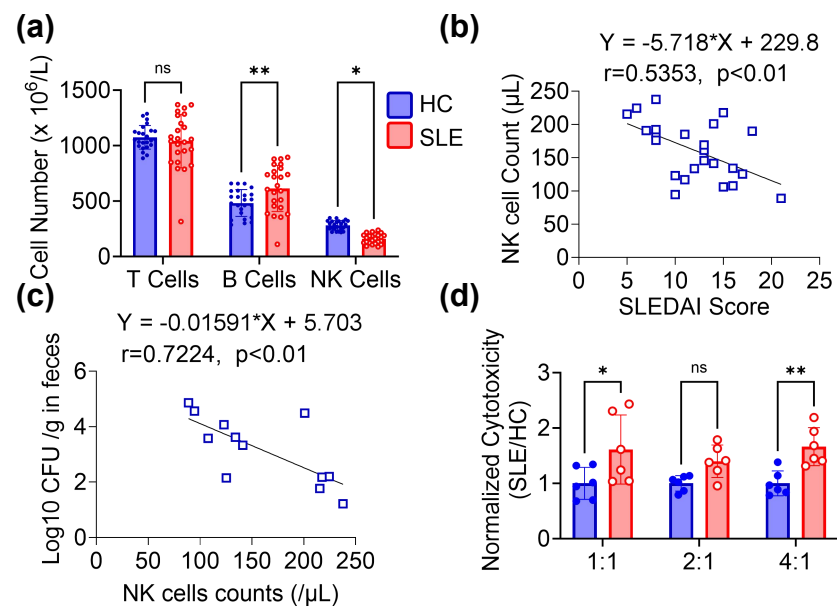

Figure S3

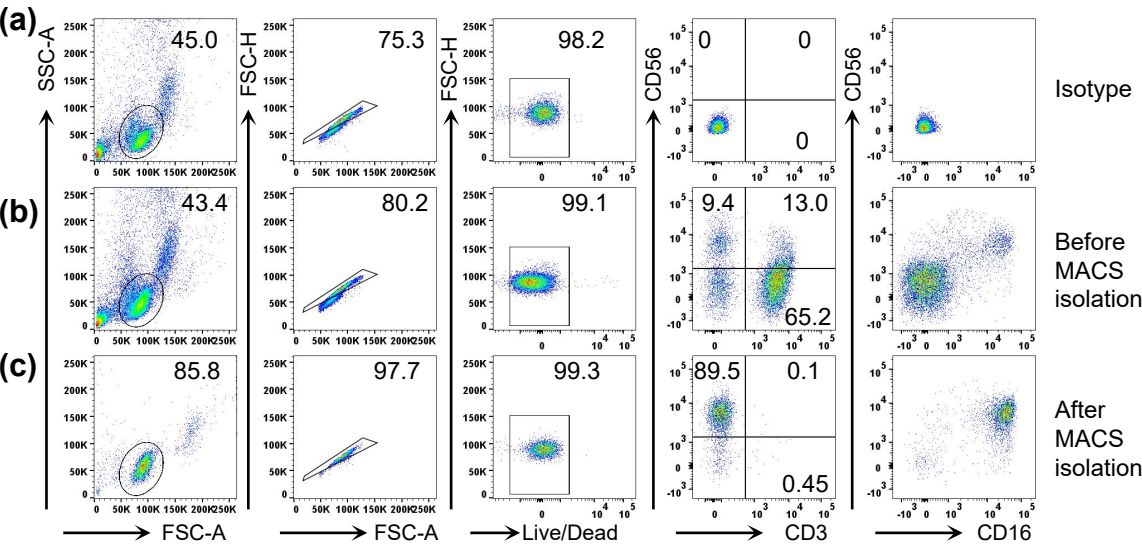

Figure S4

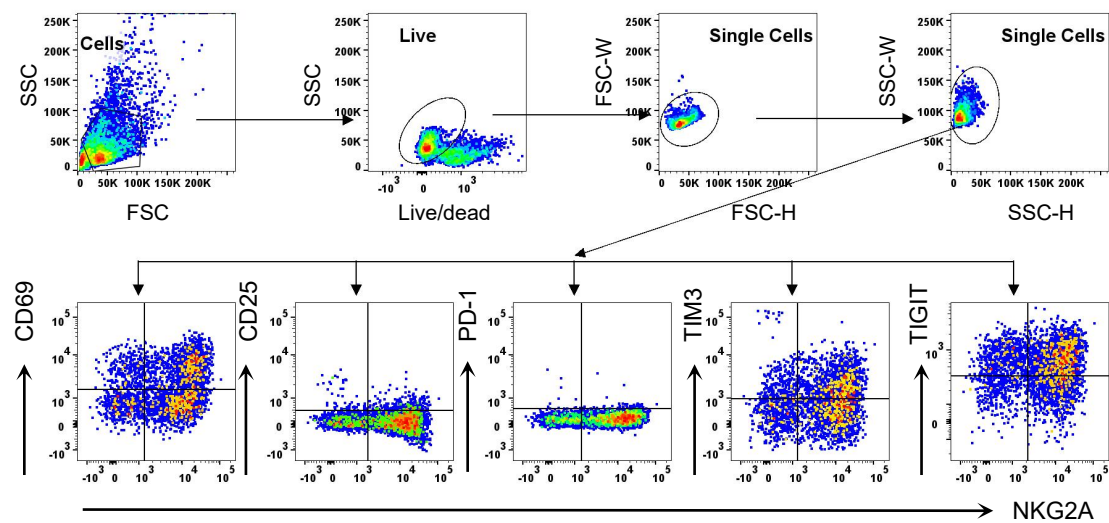

Figure S5

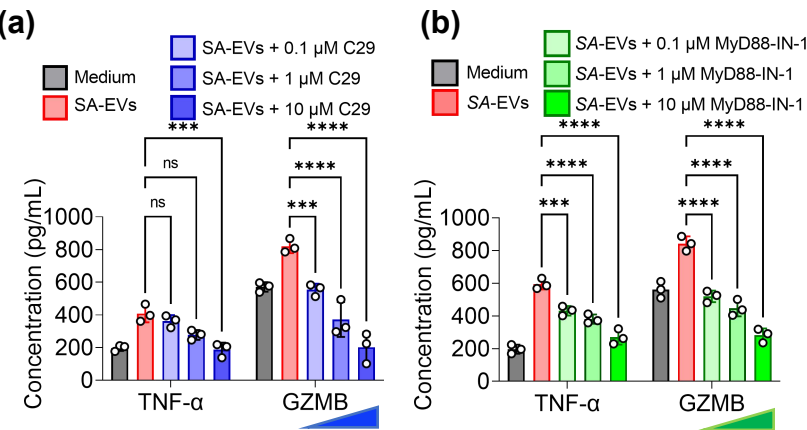

Figure S6

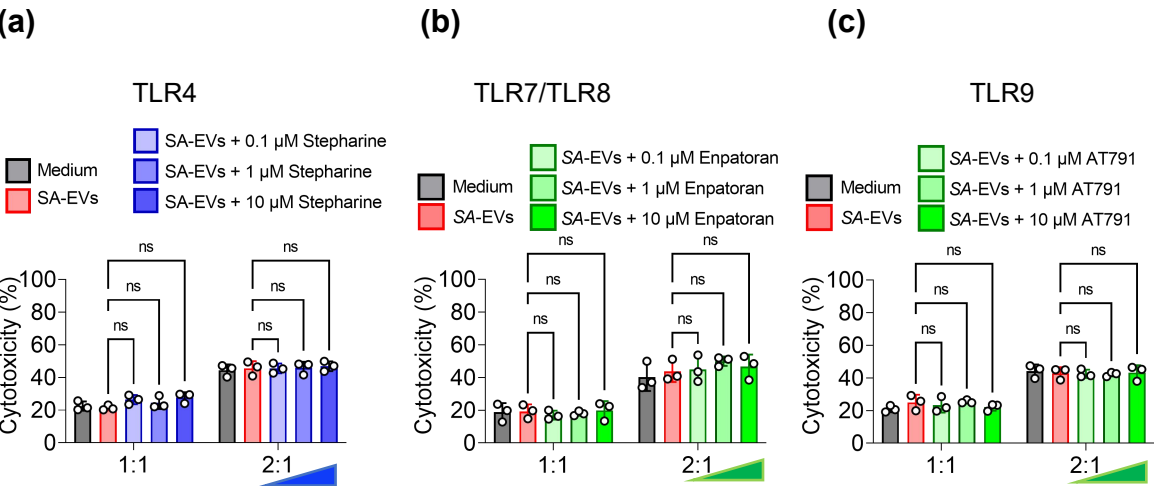

Figure S7

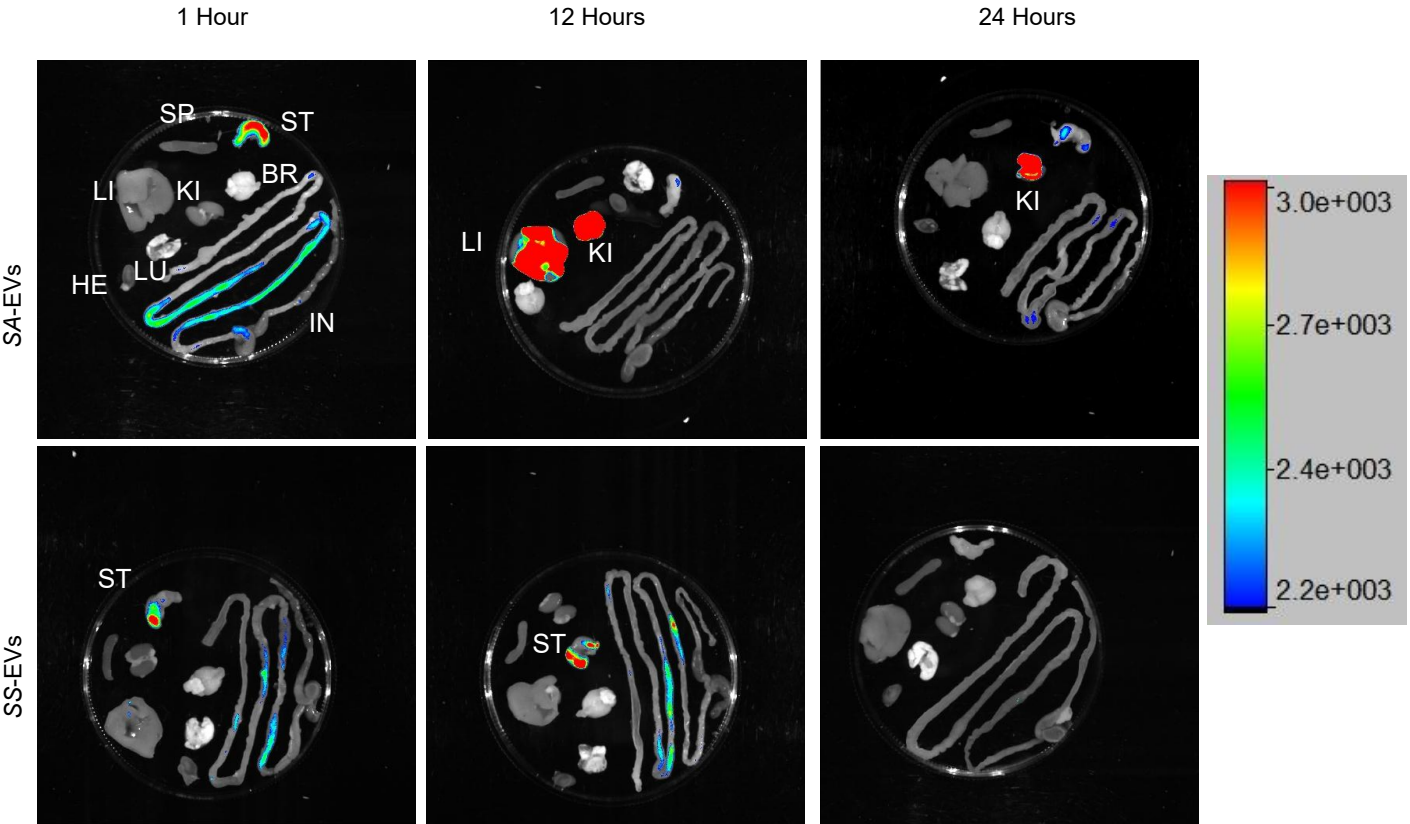

Figure S8

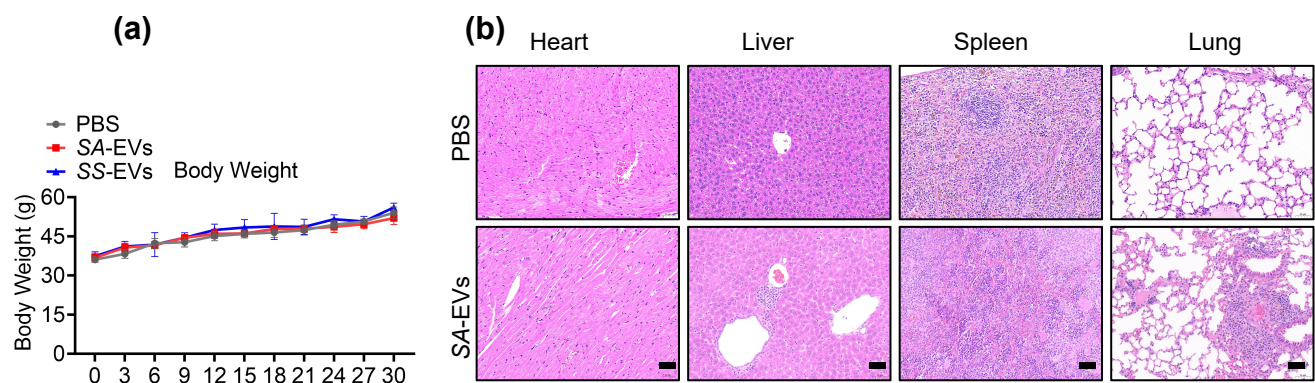

Figure S9

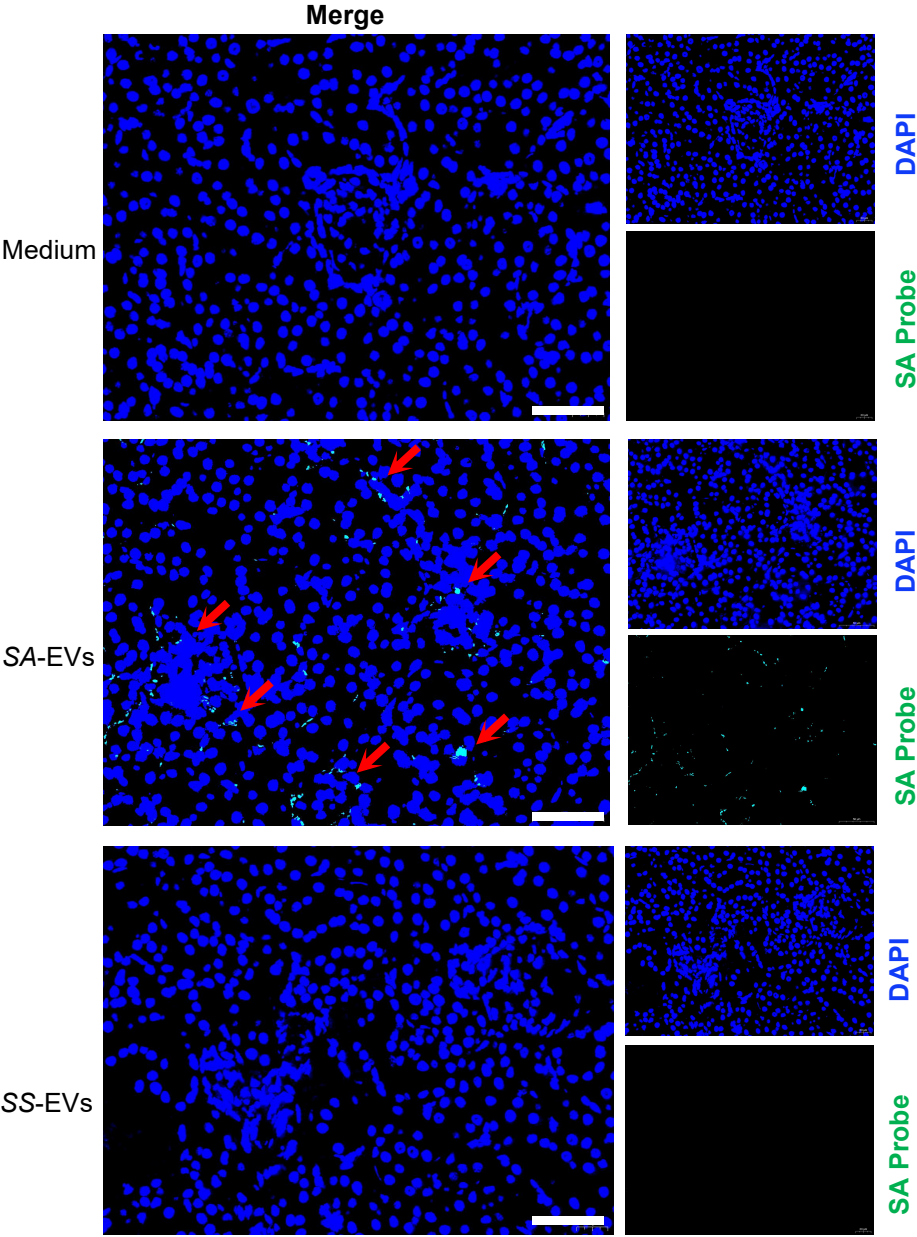

Supplement: Supplementary file 2 — Supporting Fig. 1: jev270134‐sup‐0002‐figuresS1‐S9.pdf [file JEV2-14-e70134-s002.pdf]
