# Supplementary material for: Extracellular Vesicles Derived From Streptococcus anginosus Aggravate Lupus Nephritis by Triggering TLR2‐MyD88‐NF‐κB Signalling in NK Cells
Source: J Extracell Vesicles. 2025 Jul 17;14(7):e70134. doi: 10.1002/jev2.70134 (PMC12269530; doi:10.1002/jev2.70134)
Supplement: Supplementary file 3 — Supporting Material: jev270134‐sup‐0003‐SuppMat.docx [file JEV2-14-e70134-s001.docx]

# Extracellular Vesicles derived from *Streptococcus anginosus* Aggravate Lupus Nephritis by triggering TLR2-MyD88-NF-κB signaling in NK Cells

Ying Gong ^1,2,3#^, Lingyue Jin ^1,2#^, Lina Duan ^1,2#^, Jie Xiao ^1,2#^, Yao Li ^4^, HongXia Wang ^1,2^, Haifang Wang ^1,2^, Wanying Lin ^1,2^, Yi Zhang ^1,2^, Xiufeng Gan ^1,2^, Shuyin Pang ^1,2^, Yurong Qiu ^1,2,5^, Weinan Lai ^6^, Lei Zheng ^1,2,7^, Haixia Li ^1,2^

^#^ Ying Gong, Lingyue Jin, Lina Duan and Jie Xiao contributed equally to this work.

^1.^ Department of Laboratory Medicine, Guangdong Provincial Key Laboratory of Precision Medical Diagnostics, Guangdong Engineering and Technology Research Center for Rapid Diagnostic Biosensors, Guangdong Provincial Key Laboratory of Single-cell and Extracellular Vesicles, Nanfang Hospital, Southern Medical University, Guangzhou, 510515, P. R. China.

^2^ Guangdong Provincial Clinical Research Center for Laboratory Medicine, Nanfang Hospital, Southern Medical University, Guangzhou, 510515, P. R. China.

^3^ Department of Internal Medicine, Division of Hematology, University of Maastricht, Maastricht, Netherlands.

^4^ Department of Laboratory Medicine, Foshan Hospital of Traditional Chinese Medicine, Guangzhou University of Chinese Medicine, Foshan, 528000, P.R.China.

^5^ Huayin Medical Laboratory Center Co., Ltd, Guangzhou, 510663, P.R. China

^6^ Department of Rheumatology and Immunology, Nanfang Hospital, Southern Medical University, Guangzhou, 510515, P.R. China

^7^ State Key Laboratory of Multi-organ Injury Prevention and Treatment, Nanfang Hospital, Southern Medical University, Guangzhou, China

*Correspondence author:

Prof. Dr. Haixia Li (Leader Contact)

Email: [yingchun1220@163.com](mailto:yingchun1220@163.com)

Prof. Dr. Lei Zheng

Email: [nfyyzhenglei@smu.edu.cn](mailto:nfyyzhenglei@smu.edu.cn)

Prof. Dr. Weinan Lai

Email：[Laiwn123@smu.edu.cn](mailto:Laiwn123@smu.edu.cn)

**Supplemental Material**

### DNA extraction from fecal samples

Total DNA was extracted from thawed fecal samples using the TIANGEN Stool DNA Kit (Beijing, China) in accordance with the manufacturer’s protocol. Each individually processed human fecal DNA extraction was subsequently amplified by polymerase chain reaction (PCR).

### Quantitative Real-Time PCR for Determining S. anginosus in Fecal Samples

The concentration of purified genomic DNA from S. anginosus was quantified spectrophotometrically. Using the S. anginosus ATCC 33397 1,955,308 nt genome length, we converted the measured DNA mass to exact copy number of the single-copy target gene (Costea et al., 2017). A series of 10-fold dilutions spanning ~4×10^8^ to 4 gene copies/µL was prepared in replicates. These standards were run alongside samples in each qPCR assay. S. anginosus primer forward 5'- CAAGTAGGACGCACAGTTTA-3', reverse 5'-CAAGCATCTAACATGTGTTAC-3'. Reactions were performed on a Roche Lightcycle480 PCR cycler using SYBR Green chemistry and optimized primer/annealing conditions. The standard curve gave a straight line when Ct was plotted vs. log(copy number), with R²>0.99, indicating excellent linearity. The calculated slope corresponded to ~89% efficiency, which is close to the ideal 90–110% range for SYBR assays. (Efficiency slightly <90% can occur without compromising quantitation if R² remains high.) After amplification, a melting-curve analysis was performed. We observed a single sharp peak for the stool sample S. anginosus amplicon, with no additional peaks or shoulders. No-template controls showed no amplification. Together these checks confirm that only the intended product was amplified. Each sample's Ct was converted to gene copies via the standard curve. Since the target gene is single-copy per genome, gene count ≈ cell count. We then normalized to CFU/g by accounting for extraction yield and fecal sample weight: specifically, (copies/µL from qPCR) × (total elution volume) ÷ (grams of starting feces). This yields genome equivalents per gram, i.e. CFU/g. Converting qPCR counts to CFU in this way is a recognized approach for stool microbiology (Pilarczyk-Zurek et al., 2022; Srinivasan et al., 2012 ; Zhou et al., 2022)..

### Bacterial strains and growth conditions

*Streptococcus anginosus (S. anginosus)* (Cat: ATCC 33397) was procured from the American Type Culture Collection (ATCC, Manassas, VA) and cultured at 37°C in Brain Heart Infusion (BHI) broth under shaking conditions at 200 rpm.

### Isolation of Bacterial Extracellular vesicle

For *SA*-EVs, *E.coli*-EVs and *SS*-EVs preparation, bacterial strains were cultivated at 37°C until reaching an OD600 of 0.4-0.5. *SA*-EVs isolation followed a previously described protocol (Ou et al., 2023; Wen et al., 2023). Briefly, the supernatant was centrifuged at 1,000 x g for 20 minutes, then at 10,000 x g for 30 minutes at 4°C, followed by filtration through a 0.22 μm filter. The resultant supernatant was pelleted twice via ultracentrifugation at 135,000 x g for 1.5 hours at 4°C. All centrifugation steps were performed at 4°C, using the SW 32 Ti rotor (Beckman Coulter). Isolated SA-EVs were resuspended in 500 μL PBS and stored at -80°C.

### Transmission electron microscope (TEM) for *SA*-EVs

The morphology of *SA*-EVs was analyzed using a transmission electron microscope (Hitachi H-7500), following an established protocol (Ou et al., 2023). Briefly, SA-EVs were combined with 4% paraformaldehyde, adsorbed onto carbon formvar-coated copper grids for 20 minutes, rinsed with PBS, and fixed with 1% glutaraldehyde for 2 minutes. Grids were subsequently washed with deionized water, stained with 1.5% uranyl acetate for 4 minutes, air-dried in a dark, dust-free environment, and then imaged and analyzed via TEM.

### Nanoparticle tracking analysis (NTA) for *SA*-EVs

Samples were measured using NP100 membranes with parameters of 44.5 mm and 0.64 V voltage. SA-EV samples were diluted in 0.9% NaCl and analyzed with a camera speed of 161 frames/s and a sample volume of 1 mL. The detection threshold was calibrated to maximize particle count, ensuring 10-100 red crosses with under 10% unassociated particles. Five videos of approximately 60 seconds each were recorded and analyzed using NTA software (version 2.3, NanoSight).

### Western Blot (LTA)

Lysates from bacteria, cells, and SA-EVs were prepared, and protein concentrations (20 μg/μL) determined with the BCA Protein Assay Kit (Beyotime). Samples, combined with loading buffer (Beyotime), were boiled under reducing conditions, separated via SDS-PAGE (10%, Beyotime), and transferred to PVDF membranes (MilliporeSigma, Massachusetts, USA). Primary antibodies used included lipoteichoic acid (LTA; 1:1000), anti-TLR2 (1:2000), phosphate-P65 (1:2000), P65 (1:3000), IκBα (1:1000), phosphate-IκBα (1:1000), MyD88 (1:1000), and β-Actin (1:4000).

### LTA detection by ELISA

The protein concentration of *SA*-EVs were determined byBCA. Then *SA*-EVs were lysis with RIPA buffer for 30 min on ice. Add 100 μL sample or standard to the Anti-LTA Antibody Coated Plate from LTA ELISA kit (AKR-5153, CELL BIOLABS). Each LTA unknown sample, standard and blank should be assayed in duplicate. Incubate at room temperature for 1 hour on an orbital shaker. Wash microwell strips 3 times with 250 μL 1X Wash Buffer per well with thorough aspiration between each wash. After the last wash, empty wells and tap microwell strips on absorbent pad or paper towel to remove excess 1X Wash Buffer. Add 100 μL of the diluted Biotinylated Anti-LTA antibody to each well. Incubate at room temperature for 1 hour on an orbital shaker. Wash the strip wells 3 times according to step 3 above. Add 100 μL of the diluted Streptavidin-Enzyme Conjugate to each well. Incubate at room temperature for 1 hour on an orbital shaker. Wash the strip wells 3 times according to step 3 above. Proceed immediately to the next step. Warm Substrate Solution to room temperature. Add 100 μL, of Substrate Solution to each well. Including the blank wells. Incubate at room temperature on an orbital shaker. Actual incubation time may vary from 2-30 minutes. Stop the enzyme reaction by adding 100 μL of Stop Solution into each well, including the blankwells. Results should be read immediately (color will fade over time).10. Read absorbance of each microwell on a spectrophotometer using 450 nm as the primary wavelength.

### ELISA for serum proinflammatory cytokines, ANAs and anti-double-stranded DNA (dsDNA) antibodies

The concentrations of IL-6, Granzyme B, TNF-α, IL-17, IL-22, MIP-1α, MCP-1, and CXCL8 in SLE patient serum and NK cell culture supernatants, as well as TNF, IL-6, IL-17A, IL-22, IFN-1β, and MIP-1α in mice serum, were quantified by ELISA. ANA and dsDNA antibody levels in mouse serum were measured using a mouse ANAS (ANA/ENA) ELISA kit and a mouse anti-dsDNA (total A+G+M) ELISA kit (Alpha Diagnostic), per the manufacturer’s instructions.

### SLE Mice Model

The MRL/MpJ-Faslpr/J (MRL/lpr) mouse, a well-established spontaneous SLE model (DOI: 10.1136/lupus-2021-000611), was obtained from the Shanghai SLAC Laboratory Animal (Shanghai, China) and housed under SPF conditions at the Animal Center of Nanfang Hospital, Southern Medical University. Twelve-week-old female mice were randomly assigned to receive 100 μL PBS, 20 μg/mL *SS*-EVs or *SA*-EVs gavage (n=10 per group). Following 4 weeks of treatment, mice were euthanized via pentobarbital sodium injection for sample collection. All procedures were approved by the Animal Experimentation Ethics Committee of Nanfang Hospital, Southern Medical University (No. IACUC-LAC-20231225-001) and adhered to the Institutional Animal Care and Use Committee’s animal welfare guidelines.

### Immune cells depletion in SLE Mice Model

For CD4 (Cat: BE0061), CD8 (Cat: BE0003), NK cell (Anti-NK1.1, Cat: BE0036) and B cells (anti-CD20, Cat: BE0356) depletion assays in MRL/lpr mice were followed previously study (Kruisbeek, 2001), mice were received 0.5 mg i.p. injection three days before *SA*-EVs gavage.

### *SA*-EVs Cellular Uptake Behaviors uptake assay

*SA*-EVs were labeled with the lipophilic fluorescent probe PKH67 according to the manufacturer's instructions. HK-2 cells and RPTEC/TERT1 cells were seeded in glass culture dishes for 24 hours, after which equal amounts of PKH67-labeled *SA*-EVs were added and incubated for an additional 2 hours. Cells were then washed three times with PBS and observed using a confocal laser scanning microscope (CLSM, OLYMPUS FV3000).

### Tissue distribution of *SA*-EVs

*SA*-EVs were labeled with a Deep-Red cell masking dye (Thermo Fisher Scientific) for fluorescent imaging as per the manufacturer’s instructions. Mice were gavage with Deep-Red-labeled *SA-*EVs, and fluorescence was recorded at 0, 6, and 12 hours. Mice were sacrificed, and fluorescence signals from the organs were analyzed using the Bruker fluorescence imaging system (Bruker, Germany)

### Quantitative real-time PCR (qRT-PCR)

Total RNA was extracted using the RNeasy Mini Kit (Qiagen, Hilden, Germany) according to the manufacturer’s protocol. cDNA was synthesized from 0.5 μg of total RNA using oligo dT primers, following the protocol provided in the SweScript RT I First Strand cDNA Synthesis Kit (Servicebio, Wuhan, China). Primers are listed in Table 1. For real-time PCR, 100 ng of cDNA was amplified with SYBR Green qPCR Master Mix in a Roche Light Cycler 480 apparatus (Roche). Gene expression was normalized to β-actin mRNA, and target gene expression relative to the control was calculated using the 2^−ΔΔCT^ method, expressed as fold change over average gene expression in control samples.

| Primers Table The primers for qPCR assays | | | |
| --- | --- | --- | --- |
| Gene Name | Forward Primer (5’→3’) | Reverse Primer (5’→3’) | Amplicon Size |
| *GZMB* | CCCTGGGAAAACACTCACACA | GCACAACTCAATGGTACTGTCG | 110 |
| *TNF-α* | CTCTTCTGCCTGCTGCACTTTG | ATGGGCTACAGGCTTGTCACTC | 135 |
| *IL17A* | AGATTACTACAACCGATCCACCT | GGGGACAGAGTTCATGTGGTA | 151 |
| *MIP-1α* | gaatcatgcaggtctccactg | ctcCaggtcgctgacatatttc | 272 |
| *MCP-1* | AGAATCACCAGCAGCAAGTGTCC | TCCTGAACCCACTTCTGCTTGG | 98 |
| *CXCL8* | GAGAGTGATTGAGAGTGGACCAC | CACAACCCTCTGCACCCAGTTT | 112 |
| *β-Actin* | CACCATTGGCAATGAGCGGTTC | AGGTCTTTGCGGATGTCCACGT | 135 |
| *GAPDH* | CTCATGACCACAGTCCATGC | TTCAGCTCTGGGATGACCTT | 155 |
| *S. anginosu*s primer | CAAGTAGGACGCACAGTTTA | CAAGCATCTAACATGTGTTAC | 159 |

### NK cell phenotype

NK cells were stained with fluorescently labeled antibodies (Checkpoint antibodies and TLR receptors) at appropriate dilutions for 30 minutes at 4°C in the dark. After washing in 2 mL of PBS (Gibco, Thermo Fisher), cells were centrifuged at 300 x g for 5 minutes and resuspended in 200 μL of PBS. Data were acquired on a FACS Canto II flow cytometer (BD Biosciences).

### NK cell cytotoxicity assay

NK cells isolated using negative selection as described above were used in cytotoxicity assays (Gong et al., 2021). Human proximal tubular cell (PTC) line HK-2 and RPTEC/TERT cells were used as targets to investigate NK cell killing capacity. Target cells were labeled with Cell Tracker Deep Red Dye according to the manufacturer’s protocol (Thermo Fisher Scientiﬁc, Waltham, MA, USA) the night before the cytotoxicity assay. Tumor cells were harvested using trypsinization and washing and were seeded at 2 × 10^4^ cells per well in round-bottom 96-well plates. Then, NK cells were added at various effector: target (E:T) ratios. At the same time, different concentrations of TLR receptor inhibitor were added. The total culture volume was 200 µL per well. After 30 min preincubation with mAb, NK cells were added at various effector: target (E:T) ratios. After 4 h of incubation, cells were put on ice and stained with Live/Dead Fixable Aqua (LDA) Dead Cell Stain Kit (Thermo Fisher Scientiﬁc, Waltham, CA, USA). The percentage of speciﬁc killing was calculated using the following formula:

% specific killing =% LDA positive target cells − %spontaneous LDA positive target cells × 100

% vital cells

### Protein pull-down and immunoprecipitation Assay

*SA* -EV-treated HK-2 cells were washed with PBS and lysed in cell lysis buffer for Western and IP (P0013, Beyotime, Shanghai, China) containing protease inhibitors, with all procedures performed on ice. Supernatants were incubated overnight at 4°C with either anti-LTA antibody or IgG (Cat: MSM1-1331-P1, Thermo Fisher Scientific). The immunoprecipitated protein A/G beads (Cat: 88803, Thermo Fisher Scientific) were washed thrice, eluted with 1X loading buffer (P0015A, Beyotime, China), and boiled at 99°C for 5 minutes. Beads were removed, and samples were subjected to Western blot analysis, probed with anti-TLR2 antibody, and detected by immunoblotting.

### Preparation of Renal Single Cell Suspensions

Mice were euthanized and perfused with PBS. Kidneys were excised, minced, and transferred into 4 mL of chilled RPMI 1640 with 10% FCS, 100 U/mL penicillin, and 100 μg/mL streptomycin, supplemented with 5 mg/mL Collagenase IV (Cat: 17104019, Thermo Fisher Scientific). Tissues were digested at 37°C for 45 minutes, filtered through 70 μm filters (BD Bioscience), and washed with RPMI 1640. Red blood cells were lysed in ammonium chloride solution for 5 minutes on ice and washed with RPMI 1640 medium.

### Histology, immunohistochemistry, and immunoﬂuorescence staining

Tissues were harvested and fixed in 4% paraformaldehyde (Servicebio, Guangzhou, P.R. China) for 48-72 hours. Paraffin-embedded sections (4 μm) were prepared and either H&E-stained or deparaffinized for antigen retrieval using IHC-Tek epitope retrieval solution (Servicebio) for 30 minutes. Immunofluorescence staining involved permeabilizing sections with 100% methanol, blocking with Tris-NaCl buffer, and incubating with primary antibodies (1:500) overnight at 4°C. Following washes, sections were incubated with Alexa Fluor 488-conjugated secondary antibody (Abcam) at room temperature for 1 hour. Nuclei were counterstained with DAPI (Invitrogen, Thermo Fisher), and images were captured with a Nikon ECLIPSE Ti2-U microscope (Nikon, Japan). Image analysis was performed with inForm v.2.4.8 (Akoya Biosciences) for cell population quantification (Li et al., 2023).

### Fluorescence in Situ Hybridization (FlSH)

CY3-labeled *S. anginosus* probe (Sequence: 5'-AGT TAA ACA GTT TCC AAA GCC TAC-3’ was used to detect *S. anginosus* colonization in paraffin-embedded gastric sections (Fu et al., 2024). After deparaffinization and rehydration, specimens were treated with 0.2 N HCl and Proteinase K for 10 min for each step. Following incubation with blocking bufer on 55 ℃ for 2 hours, CY3-labeled *S. anginosus* probe (1:50 in 35% hybridization buffer, pre-heated at 88 'C for 3 min before use) was added and hybridized in a dark, humid chamber at 42'C overnight. Specimens were washed in wash solution (20 mL Tris-HCl, pH=7.2: 40 mM NaCl and mounted with DAPl-antifade solution (P36931. Thermo Fisher Scientific). Images were acquired with a fluorescent microscope (Nikon).

### LC-MS/MS Analysis

LC-MS/MS experiments were performed on a Q Exactive HF-X mass spectrometer that was coupled to Easy nLC1200 (Thermo Scientific). Peptide was first loaded to a trap column (100μm*20mm, 5μm，C18，Dr. Maisch GmbH, Ammerbuch, Germany) in buffer A (0.1% Formic acid in water). Reverse-phase high-performance liquid chromatography (RP-HPLC) separation was performed using a self-packed column (75 μm × 150 mm; 3 μm ReproSil-Pur C18 beads, 120 Å, Dr. Maisch GmbH, Ammerbuch, Germany) at a flow rate of 300 nL/min. The RP−HPLC mobile phase A was 0.1% formic acid in water, and B was 0.1% formic acid in 95% acetonitrile. The gradient was set as following: 2%–4% buffer B from 0 min to 2min, 4% to 30% buffer B from 2 min to 47 min, 30% to 45% buffer B from 47 min to 52 min, 45% to 90% buffer B from 52 min to 54 min, 90% buffer B kept till to 60min. MS data was acquired using a data-dependent top20 method dynamically choosing the most abundant precursor ions from the survey scan (350–1800 m/z) for HCD fragmentation. A lock mass of 445.120025 Da was used as internal standard for mass calibration. The full MS scans were acquired at a resolution of 60,000 at m/z 200, and 15,000 at m/z 200 for MS/MS scan. The maximum injection time was set to for 50 ms for MS and 25 ms for MS/MS. Normalized collision energy was 28 and the isolation window was set to 1.6 Th. Dynamic exclusion duration was 30 s.

### Sequence Database Searching and Data Analysis

The MS data were analyzed using MaxQuant software version 1.6.1.0. MS data were searched against the UniProtKB *Streptococcus anginosus* [1328]20946-20241107.fasta. The trypsin was selected as digestion enzyme. The maximal two missed cleavage sites and the mass tolerance of 4.5 ppm for precursor ions and 20 ppm for fragment ions were defined for database search. Carbamidomethylation of cysteines was defined as fixed modification, while acetylation of protein N-terminal, oxidation of Methionine were set as variable modifications for database searching. The database search results were filtered and exported with <1% false discovery rate (FDR) at peptide-spectrum-matched level, and protein level, respectively.

**Supplemental Figure Legends**

**Supplemental Figure 1.** Streptococcus anginosus is enriched in SLE patients and positively correlates with disease severity. Relative abundance of S. anginosus is significantly elevated in fecal samples from SLE patients compared to healthy controls (HC) (N = 12). (b) Spearman’s correlation between ΔCt values of S. anginosus and SLEDAI scores in SLE patients. Data are presented as mean ± SD, with individual data points representing biological replicates (average of technical duplicates). Statistical comparisons were performed using t test. p < 0.05 (*).
**Supplemental Figure 2.** Reduced absolute NK cell counts and elevated cytokine production in the peripheral blood of SLE patients. (a) Absolute counts of TBNK lymphocyte subsets in peripheral blood of HC and SLE patients (N = 24). (b) Correlation between NK cell counts and SLEDAI scores in SLE patients. (c) Correlation between NK cell counts and S. anginosus CFU counts in SLE patients. (d) Normalized NK cell cytotoxicity against K-562 target cells from HC and SLE patients, corresponding to Figure 1f. Data are presented as mean ± SD, with individual data points representing biological replicates (average of technical duplicates). Statistical comparisons were performed using one-way ANOVA with Tukey’s post hoc test. p < 0.05 (*); p < 0.01 (**); p < 0.001 (***); p < 0.0001 (****); ns: not significant.

**Supplemental Figure 3.** Gating strategy and purity of NK cell following MACS sorting.
(a) The Isotype control. (b) PBMCs prior to MACS isolation. (c) NK cells post-MACS purification.

**Supplemental Figure 4.** The gating strategy of NK cell phenotyping.

**Supplemental Figure 5.** *SA*-EVs stimulate NK cells secrete TNF-α and granzyme B, which is abrogated by TLR2-MyD88 inhibitors. Cytokine production of NK cells co-cultured with RPTEC/TERT1 cells in the presence with TLR2 inhibitor (C29) (a) or MyD88 inhibitor (MyD88-IN-1) (b) with or without 1 μg/mL *SA*-EVs coculture for 48 h (N=3). Supernatant concentration of TNF-α and granzyme B were detected by ELISA. Data are shown as mean ± SD, with dots representing individual donors (average of technical duplicates). Statistical differences between groups were determined using one-way ANOVA with Tukey post-tests. p < 0.001 (***); p < 0.0001 (****); ns: not significant.
**Supplemental Figure 6.** TLR4, TLR7 and TLR9 inhibitors inhibition does not affect NK cell cytotoxicity.

Cytotoxicity of NK cells against RPTEC/TERT1 cells in presence with TLR4 inhibitor (Stepharine) (a)，TLR7 inhibitor (Enpatoran) (b) or TLR9 inhibitor (AT791) (c) with or without 1 μg/mL *SA*-EVs coculture for 48 h (N=3). Data is shown as mean ± SD, with dots representing individual donors (average of technical duplicates). Statistical differences between groups were determined using one-way ANOVA with Tukey post-tests. p < 0.001 (***); p < 0.0001 (****); ns: not significant.
**Supplemental Figure 7.** Fluorescence images of major organs from sacrificed mice at different time points (1 hour, 12 Hours and 24 Hours) post-treatment, including heart (HE), liver (LI), spleen (SP), lung (LU), kidneys (KI),Stomach (ST), Brain (BR) and Intestine(IN).

**Supplemental Figure 8.** Body weight monitoring and histopathology of visceral organs in SA-EV–treated MRL/lpr mice. Body weight changes in MRL/lpr mice during the course of treatment. (b) Representative H\&E-stained sections of visceral organs from treated MRL/lpr mice. Data is shown as mean ± SD.

**Supplemental Figure 9** Representative FISH images of renal tissue sections from mice receiving oral S. anginosus EVs (1 month post-treatment). Blue: nuclei; green: S. anginosus-specific probe. Scale bars, 50 μm.

Reference:

Costea, P. I., Zeller, G., Sunagawa, S., Pelletier, E., Alberti, A., Levenez, F., Tramontano, M., Driessen, M., Hercog, R., Jung, F. E.*, et al.* (2017). Towards standards for human fecal sample processing in metagenomic studies. *Nature biotechnology, 35 (11)*, 1069-1076. <https://doi.org/10.1038/nbt.3960>

Fu, K., Cheung, A. H. K., Wong, C. C., Liu, W., Zhou, Y., Wang, F., Huang, P., Yuan, K., Coker, O. O., Pan, Y.*, et al.* (2024). Streptococcus anginosus promotes gastric inflammation, atrophy, and tumorigenesis in mice. *Cell, 187 (4)*, 882-896. <https://doi.org/10.1016/j.cell.2024.01.004>

Gong, Y., Klein Wolterink, R. G. J., Gulaia, V., Cloosen, S., Ehlers, F. A. I., Wieten, L., Graus, Y. F., Bos, G. M. J., and Germeraad, W. T. V. (2021). Defucosylation of Tumor-Specific Humanized Anti-MUC1 Monoclonal Antibody Enhances NK Cell-Mediated Anti-Tumor Cell Cytotoxicity. *Cancers (Basel), 13 (11)*, 2579. <https://doi.org/10.3390/cancers13112579>

Kruisbeek, A. M. (2001). In vivo depletion of CD4- and CD8-specific T cells. *Curr Protoc Immunol, Chapter 4*, Unit 4.1. <https://doi.org/10.1002/0471142735.im0401s01>

Li, J., Wu, C., Hu, H., Qin, G., Wu, X., Bai, F., Zhang, J., Cai, Y., Huang, Y., Wang, C.*, et al.* (2023). Remodeling of the immune and stromal cell compartment by PD-1 blockade in mismatch repair-deficient colorectal cancer. *Cancer cell, 41 (6)*, 1152-1169.e1157. <https://doi.org/10.1016/j.ccell.2023.04.011>

Ou, Z., Situ, B., Huang, X., Xue, Y., He, X., Li, Q., Ou, D., He, B., Chen, J., Huang, Y.*, et al.* (2023). Single-particle analysis of circulating bacterial extracellular vesicles reveals their biogenesis, changes in blood and links to intestinal barrier. *J Extracell Vesicles, 12 (12)*, e12395. <https://doi.org/10.1002/jev2.12395>

Pilarczyk-Zurek, M., Sitkiewicz, I., and Koziel, J. (2022). The Clinical View on Streptococcus anginosus Group - Opportunistic Pathogens Coming Out of Hiding. *Front Microbiol, 13*, 956677. <https://doi.org/10.3389/fmicb.2022.956677>

Srinivasan, V., Gertz, R. E., Jr., Shewmaker, P. L., Patrick, S., Chitnis, A. S., O'Connell, H., Benowitz, I., Patel, P., Guh, A. Y., Noble-Wang, J.*, et al.* (2012). Using PCR-based detection and genotyping to trace Streptococcus salivarius meningitis outbreak strain to oral flora of radiology physician assistant. *PloS one, 7 (2)*, e32169. <https://doi.org/10.1371/journal.pone.0032169>

Wen, M., Wang, J., Ou, Z., Nie, G., Chen, Y., Li, M., Wu, Z., Xiong, S., Zhou, H., Yang, Z.*, et al.* (2023). Bacterial extracellular vesicles: A position paper by the microbial vesicles task force of the Chinese society for extracellular vesicles. *Interdiscip Med, 1 (3)*, e20230017. <https://doi.org/https://doi.org/10.1002/INMD.20230017>

Zhou, C. B., Pan, S. Y., Jin, P., Deng, J. W., Xue, J. H., Ma, X. Y., Xie, Y. H., Cao, H., Liu, Q., Xie, W. F.*, et al.* (2022). Fecal Signatures of Streptococcus anginosus and Streptococcus constellatus for Noninvasive Screening and Early Warning of Gastric Cancer. *Gastroenterology, 162 (7)*, 1933-1947.e1918. <https://doi.org/10.1053/j.gastro.2022.02.015>
